# Bioactive glass-based core-shell nanoparticles: Multifunctional platforms for controlled drug release and biomedical applications

**DOI:** 10.1016/j.mtbio.2025.102617

**Published:** 2025-12-01

**Authors:** Arthur M. Gabriel, Andrada-Ioana Damian-Buda, Fernanda M. Brugnari, Emerson R. Camargo, Aldo R. Boccaccini

**Affiliations:** a Institute of Biomaterials, University of Erlangen-Nuremberg, 91058, Erlangen, Germany; b Department of Chemistry, Federal University of São Carlos, 13565-905, São Carlos, Brazil

**Keywords:** Bioactive glass, Core shell, Controlled drug release, Biomaterials

## Abstract

Core-shell engineered nanoparticles have emerged as multifunctional platforms for biomedical applications by enabling precise spatial control over their structure, controlled release, and interaction with biological tissues. Among the core materials, bioactive glasses offer unique advantages over conventional silica due to their intrinsic bioactivity and the release of therapeutic ions. This review provides a comprehensive analysis of core-shell nanostructures that use bioactive glass as the core, and shells composed of a wide range of inorganic and polymeric materials, engineered to control their release capabilities and biological performance. The composition and synthesis strategies of bioactive glass nanoparticles are discussed. The types of shell materials are also evaluated, highlighting their physicochemical roles in modulating drug diffusion, stability, degradation, and biological targeting. Inorganic, natural, and synthetic polymeric shells are discussed, focusing on how they can be used to tailor the properties of the nanoparticles. Key challenges related to in vivo performance assays, immunological responses, degradation behavior, and translational barriers, outlining future directions for clinical implementation, are also explored. By integrating the structural functionalities of bioactive glasses with advanced surface engineering, the core-shell structures discussed in this review represent a versatile, highly tunable and customizable strategy for next-generation biomedical therapies.

## Highlights

• First comprehensive review on bioactive glass core–shell nanoparticles.
• BGN cores provide ion release, osteogenesis, angiogenesis, and antibacterial effects.
• Inorganic and polymeric shells enable stability, controlled release, and targeting.
• Core–shell BGNs are promising for drug delivery and regenerative medicine.
• Challenges and perspectives for in vivo translation and clinical applications.

## Graphical abstract

**Figure:**
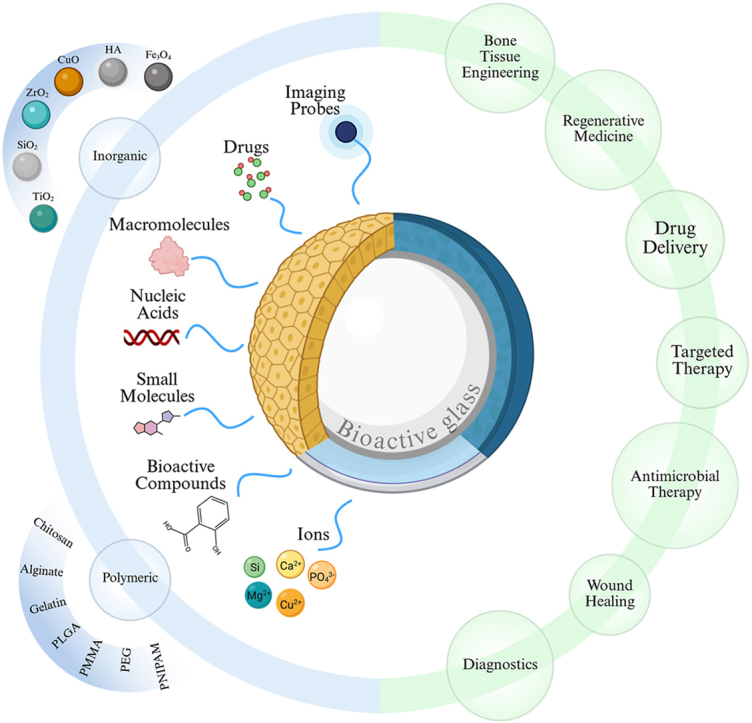

## 1 Introduction

In recent years, nanomaterials have played a leading role in the modern biomedical technology landscape due to their size, high surface area, and tunable surface chemistry [1,2]. Their properties enable applications such as controlled release systems, tissue regeneration, imaging, and antimicrobial therapies [3]. Systems composed of silica, gold, and iron oxide nanoparticles have become attractive due to their structural stability and ease of functionalization [2]. The possibility of offering responsiveness to stimuli, biocompatibility, and therapeutic efficiency is a significant advantage over traditional bulk biomaterials [1].

Silicon dioxide (SiO_2_), or silica, is highly abundant on Earth, comprising roughly 59–60 % of the continental crust, whereas in the human body silicon is present in trace amounts, approximately 1–2 g total in an adult (≈20–30 mg/kg body weight), mostly in soluble form such as orthosilicic acid rather than as crystalline silica [4,5]. Silica nanoparticles (SiNPs) have gained significant attention due to their low cost, high surface area, stability, and the presence of surface silanol groups that enable versatile functionalization [6,7]. In addition to being slowly biodegradable and largely biocompatible, in given concentrations these structures are suitable for biotechnological and biomedical applications [8,9]. A key application of SiNPs is in drug delivery, first reported by Vallet-Regí et al. [10], leading to extensive research into their use for targeted and controlled therapy against diseases such as cancer, Alzheimer's, and infections. They also serve as multifunctional platforms for imaging, due to their ability to carry fluorescent or magnetic agents [[11], [12], [13], [14], [15]]. For instance, mesoporous silica nanoparticles (MSNs), functionalized with a pH-sensitive polyacrylic acid gate and a targeting lectin were developed by Martínez-Carmona et al. [16], aiming to enable selective delivery of doxorubicin to osteosarcoma cells. This system showed enhanced cellular uptake, pH-triggered drug release, and significantly higher cytotoxicity toward tumor cells compared to free drug or non-targeted carriers [16]. Similarly, Lu et al. [17] designed MSNs coated with polydopamine (PDA) and chelated with Mn^2+^ ions for dual magnetic resonance imaging (MRI)/fluorescence imaging and combined photothermal–photodynamic cancer therapy. This approach enabled efficient tumor imaging and significant in vivo antitumor effects under near-infrared laser irradiation [17]. The versatility of SiNPs lies in their tunable properties, including size, porosity, surface chemistry, and their ability to support the design of structures like nonporous, mesoporous, and core-shell (CS) nanoparticles [18,19]. However, SiNPs generally lack bioactivity (biological reactivity), a critical feature for applications such as tissue regeneration [20,21].

Bioactive glasses (BGs) are non-crystalline inorganic materials based on silicate, borate or phosphate compositional systems. BGs have emerged as key inorganic materials due to their ability to form hydroxyapatite (HA) layers in physiological environments and to release ions that stimulate bone growth [22]. To further enhance the performance of traditional BGs, BG nanoparticles (BGNs) have been developed, combining nanoscale advantages, mainly high surface area to volume ratio, tunable ion release, enhanced cellular interactions and versatility for use in composite systems. Unlike silica, BGNs can release biologically active ions (e.g., Ca^2+^, SiO_4_^4−^, PO_4_^3−^, and doped species like Cu^2+^, Zn^2+^, Sr^2+^) that can induce various therapeutic effects, such as antimicrobial activity, osteogenesis, angiogenesis, and immunomodulation [20,23,24]. In this regard, copper-doped mesoporous bioactive glass nanoparticles (MBGNs) were fabricated by Bari et al. [25] and showed effective antibacterial activity against *E. coli, S. aureus,* and *S. epidermidis*. At the same time, MBGNs were able to reduce and disrupt biofilm matrix, while showing bioactivity and a great potential as multifunctional therapeutic agents against infections [25]. Zheng et al. [26] developed antioxidant Ce-doped MBGNs that demonstrated antioxidant properties by effectively reducing oxidative stress in cells and anti-inflammatory effects by downregulating proinflammatory gene expression and pro-osteogenic activities by suppressing pro-osteoclastogenic responses [26]. The ion-exchange-driven bioactivity makes MBGNs very attractive materials for the formulation of advanced drug delivery systems that, together with the possibility of tailoring degradation rate, bioactivity and therapeutic function, combine regeneration and therapeutic modulation with controlled release, thus achieving synergistic therapeutic effects [22,27].

Despite their well-recognized bioactivity, BGNs are often limited by uncontrolled dissolution and rapid ion release once exposed to physiological environments. This behavior can lead to premature depletion of therapeutic ions, instability of the material surface, and even cytotoxic effects at high concentrations [28,29]. To overcome these challenges, the design of CS architectures has emerged as a promising strategy, in which a protective shell modulates the interaction between the BG core and the surrounding medium. By tailoring the composition, thickness, and permeability of the shell, it becomes possible to achieve a more gradual and sustained ionic release profile, while preserving the intrinsic bioactivity of the BG core [30].

CS nanostructure architectures are modular and highly tunable platforms for biomedical applications, combining distinct core and shell functionalities with precise control over release kinetics, surface interactions, and biological stability [19,31,32]. A CS material was developed using CeO_2_ and MSNs by Șen Karaman et al. [33] to carry capsaicin, which showed significant in vitro and in vivo antibacterial efficacy against *E. coli*. This design enhanced the cytocompatibility of capsaicin, and effectively reduced the therapeutic dose, while ensuring safety for mammalian cells, making it a promising candidate for combating bacterial infections with reduced toxicity [33].

In the case of BGN-based cores, the shell layer plays a critical role in modulating ion diffusion, extending release duration, and providing specific interactions with biological targets [34]. These shells can be composed of inorganic or polymeric materials. Furthermore, the use of smart materials enables responsiveness to external stimuli such as pH, temperature, or enzymes, enabling smart delivery systems that release their cargo only under specific pathological conditions [19,35,36]. Herrmann et al. [37] fabricated responsive core-shell-shell nanocarriers using iron oxide, MSNs, and a pH sensitive polymer. This system was able to release enrofloxacin specifically in acidic environments and be directed by magnetic fields, thus avoiding premature release and enhancing the local therapeutic efficacy [37].

In light of these advances, this review aims to provide a comprehensive and critical overview of the state-of-the-art of CS nanomaterial systems that use BGNs as the core. By combining the therapeutic ion and drug delivery capabilities of BGNs with a wide range of shell materials (such as inorganic or polymeric materials), these hybrid nanoarchitectures offer a promising alternative for the fabrication of multifunctional platforms capable of drug delivery, tissue regeneration and immunomodulation. Thus, the first part of this review will introduce the advantages of BGNs as core material, followed by a detailed discussion on the combination of BGNs as core with different shell materials. Special emphasis will be placed on the synthesis methods and the biofunctional mechanisms associated with these structures, with a particular attention on how different shell materials can modulate the biological interface and therapeutic performance. Finally, the last part of this paper will present the current challenges and future perspectives of these promising BGN-based hybrid platforms.

The bibliographic research was conducted using three major scientific databases: Scopus, Web of Science (WoS), and PubMed. The search strategy employed Boolean operators, controlled vocabulary, and truncation symbols where appropriate. The following keywords and search strings were applied:

Scopus search string:

TITLE-ABS-KEY(("core-shell" OR "core shell" OR "hybrid nanoparticle∗" OR "coated nanoparticle∗") AND ("bioactive glass" OR "mesoporous bioactive glass" OR BGN OR MBGN) AND ("drug delivery" OR "controlled release" OR "tissue engineering" OR "regenerative medicine" OR "antibacterial" OR "therapeutic"))

Web of Science (WoS) search string:

TS=(("core-shell" OR "core shell" OR "hybrid nanoparticle∗" OR "coated nanoparticle∗") AND ("bioactive glass" OR "mesoporous bioactive glass" OR BGN OR MBGN) AND ("drug delivery" OR "controlled release" OR "tissue engineering" OR "regenerative medicine" OR "antibacterial" OR "therapeutic"))

PubMed search string:

(("core-shell"[tiab] OR "core shell"[tiab] OR "hybrid nanoparticle∗"[tiab] OR "coated nanoparticle∗"[tiab]) AND ("bioactive glass"[tiab] OR "mesoporous bioactive glass"[tiab] OR BGN[tiab] OR MBGN[tiab]) AND ("drug delivery"[tiab] OR "controlled release"[tiab] OR "tissue engineering"[tiab] OR "regenerative medicine"[tiab] OR "antibacterial"[tiab] OR "therapeutic"[tiab]))

Filters: Publications in English; only articles and reviews; timeframe: last 15 years (2010–2025). Conference abstracts and non-peer-reviewed materials were excluded. The articles were read individually and judged whether they fit the topic.

While several previous reviews have discussed the synthesis and applications of BGNs or polymer–ceramic hybrid systems, none have systematically analyzed CS architectures in which BGNs serve as functional cores combined with inorganic or polymeric shells. This review aims to bridge that gap by providing a comparative and critical overview of BGN-based CS nanostructures, emphasizing how shell composition and fabrication strategy influence ion release, mechanical stability, and biological response. To better contextualize the novelty of this work, Table 1 compares the present review with previous articles focused on BGNs or hybrid nanocarriers. While earlier reviews primarily discussed synthesis and general applications, none of them provided a systematic comparison of CS architectures involving BG cores.

**Table 1:** Comparison between the present review and previous review articles on bioactive glass and hybrid nanocarrier systems.

Ref	Main Focus	Materials discussed	Limitations/missing aspects	Novelty of the present review
[38]	The biological response to ionic dissolution products from BGs and glass-ceramics; It specifically investigates the effect of released ions (Si(OH)_4_, Ca^2+^, Sr^2+^, Zn^2+^, Mg^2+^, and Cu^2+^) on osteogenesis and angiogenesis	Silicate BGs (pure and ion-doped), phosphate-based glasses, and glass-ceramics	Focus only on the biological effect of the particles, not on the nanoparticle architecture	Focused analysis on a specific fabrication strategy designed to precisely control the ion release and biological responses
[39]	Current clinical applications, and future challenges of BGs	Various BG forms, including 45S5, commercial particulates, coatings, 3D-printed scaffolds and hybrids	Lacks a specific, in-depth analysis of CS nanostructures as a distinct class of materials	In-depth review of a specific advanced material design (BGN-based CS) and its performance
[40]	BG coatings applied to the surface of metallic implants	BG coatings on metallic substrates; Techniques discussed include thermal spraying, enameling, sol-gel, electrophoretic deposition and laser cladding	Limited to macroscopic coatings on implant surfaces; Does not discuss the use of nanoparticles in injectable systems or composite fillers	This work analyzes a completely different material form (discrete nanoparticles) rather than the macroscopic surface coatings
[41]	An analysis of organic/inorganic hybrids based on BGs, focusing on polymer design and chemistry to create materials mixed at the molecular level	Class I (weak interactions) and Class II (covalent bonds) hybrids; Discusses polymers such as PCL, PEG, PDMS, gelatin and chitosan within a sol-gel matrix	Focuses exclusively on molecular-level hybrids, which are structurally distinct from nanocomposite CS systems	This work reviews multi-phasic CS nanocomposite systems
[42]	BG-polymer nanocomposites, where BGNs are dispersed as a filler within a polymer matrix	Natural and synthetic polymer matrices (e.g., alginate, chitosan, PCL, PLA) containing BGNs as a reinforcing phase	Focuses on composite mixtures/blends, which is structurally distinct from CS architectures, as each BGN is individually covered by a shell material	This work focuses on a more advanced nano-architecture (CS) offering superior control over surface properties and release kinetics, rather than the nanocomposite blends
This work	CS nanoparticles with BG cores	Inorganic and polymeric shells		First comprehensive review dedicated to CS architectures with BGN cores; Includes comparative analysis of fabrication methods, release mechanisms, and biological outcomes
BG – bioactive glass, BGNs – bioactive glass nanoparticles, CS – core-shell, PLA – polylactic acid, PCL – polycaprolactone, PEG – polyethylene glycol, PDMS – polydimethylsiloxane.				

## 2 Bioactive glass nanoparticles as core of core-shell nanosystems

The use of BGNs as a core in CS systems is based on their several inherent properties. Their intrinsic bioactivity and their ability to chemically bind to a host tissue and induce regeneration set BGNs apart from more inert materials such as pure silica [[43], [44], [45], [46]]. Added to these advantages are the therapeutic, antibacterial, anti-inflammatory and osteogenic effects that can be activated by the BGN ability to release controlled and localized ions and molecules [45,[47], [48], [49], [50]]. Furthermore, BGNs exhibit biodegradability [51], high surface area, and porosity [52]. Combined with the outstanding chemical functionalization versatility [44], these properties make BGNs strong candidates for the fabrication of CS nanostructures tailored to specific clinical needs, serving both as a scaffold and a secondary source of therapeutic function [44,49,53].

**Fig. 1:**
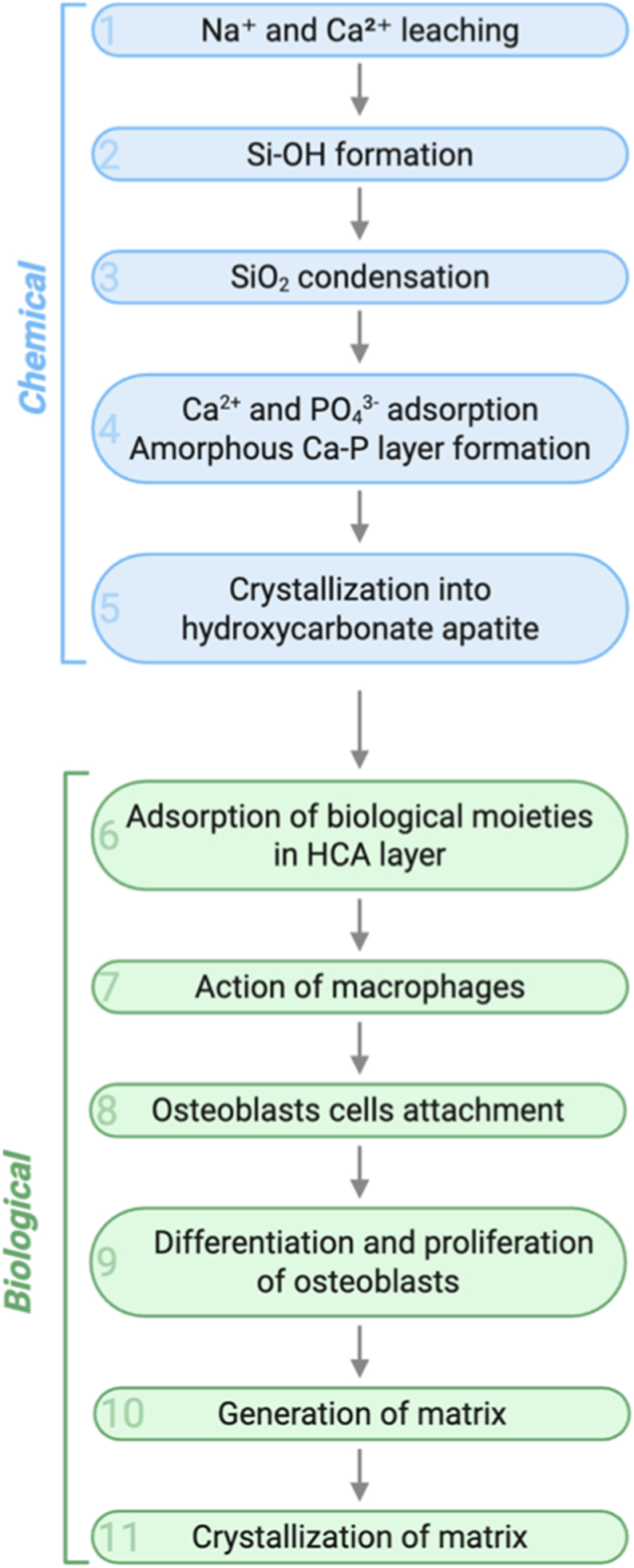
Representation of the eleven stages taking place on the surface of BGs when in contact with physiological fluids [69]. Created in BioRender. Gabriel, A. (2025) https://BioRender.com/ mjvwbh3.

The ability to bind to bone and soft tissue, e.g. for drug or growth factor delivery, makes BGs highly attractive biomaterials. This occurs through a well-understood eleven-stage reaction when exposed to physiological fluids [54,55]. Five of these stages are considered chemical reactions, and the other six involve biological and cellular processes, as shown in Fig. 1. The process begins with the leaching of Na^+^ and Ca^2+^ ions, which raises the pH at the surface. Then, silanol groups (Si-OH) are formed due to the dissolution of the SiO_2_ network. These groups condense to form a silica-rich layer, forming a porous layer of hydrated silica gel, which subsequently acts as a template for the nucleation of a calcium phosphate (Ca-P) phase. Then, calcium and phosphate ions from the surrounding fluid (and the material) are adsorbed, leading to the formation of an amorphous calcium phosphate layer. The final chemical step involves the crystallization of this amorphous calcium phosphate together with carbonate ions into hydroxycarbonate apatite (HCA), a mineral chemically and structurally similar to bone. After the HCA layer forms, biological molecules adsorb onto the mineral layer. Macrophages interact with the biomaterial, initiating an immune response and releasing inflammatory and regenerative signals [[56], [57], [58], [59]]. The bioactive surface then promotes the adhesion of osteoblasts, which proliferate and differentiate, generating an extracellular organic matrix rich in collagen and bone proteins that crystallize and form new bone tissue. This structure enables BGs to bond directly and strongly to bone tissue [60,61].

In parallel with the formation of apatite, BGs also have biological effects by releasing ions with therapeutic effects. These ions, such as soluble silica species [e.g., orthosilicic acid], Ca^2+^, PO_4_^3−^, and additional dopants, can modulate several cellular processes including proliferation, differentiation, gene expression, and angiogenesis [[62], [63], [64], [65]]. For example, soluble silica species (e.g., Si(OH)_4_) can promote osteoblast differentiation and extracellular matrix (ECM) production, while Ca^2+^ play a key role in intracellular signaling pathways. It is also worth mentioning that the ion release profile is highly dependent on the compositional and textural properties of the particle, such as size, surface area, and porosity [[66], [67], [68]]. This property makes the material a tunable platform for use as a controlled release device for targeted biological responses.

BG was discovered in 1969 by Prof. L. L. Hench. The silicate material, now known as 45S5 Bioglass, consists of 45 % SiO_2_, 24.5 % CaO, 24.5 % Na_2_O and 6 % P_2_O_5_ (wt%) [62,[69], [70], [71]]. Numerous variations of BGs have been reported, with different compositions (e.g., S53P4, 13–93) developed over the years to tailor degradation rates, bioactivity levels, mechanical strength, and ion release profiles. These materials include phosphate-, borate- or silicate-based glasses and often feature the use of substituent elements, such as Zn^2+^, Sr^2+^, Cu^2+^ or Ag^+^, capable of conferring specific therapeutic effects [72,73]. For instance, it has been shown that the incorporation of Sr^2+^ can improve osteogenic differentiation, while Cu^2+^ and Ag^+^ can impart angiogenic and antimicrobial properties, respectively [[74], [75], [76], [77]]. These compositional modifications have been an important tool for enhancing the biological activity of BGs. Thus, it is not surprising that in recent years, the development of BGNs has led to numerous applications, including soft tissue engineering and in cancer therapy, in addition to the traditional applications in bone repair and dental materials. The ongoing evolution of BGNs reflects their high compositional flexibility and ability to be engineered for multifunctional bioactive responses [61,63,78,79].

The synthesis method of BGNs has a strong influence on their final structure, composition and bioactivity. Traditional techniques such as melt-quenching can be used to fabricate bulk or macroparticulate BGs, but they have limitations in terms of nanometric production. In contrast, the sol–gel synthesis technique has become predominant for obtaining BGNs because it can produce highly porous and highly homogeneous nanomaterials at relatively low temperatures [80,81]. The synthesis of BGNs is most commonly achieved through the sol–gel process, in which silica precursors, such as tetraethyl orthosilicate (TEOS), undergo hydrolysis and condensation reactions to form a three-dimensional silica network. During this process, calcium and phosphate salts are incorporated into the sol, enabling the homogeneous distribution of these ions in the resulting glass matrix. The reaction is typically conducted in ethanol–water mixtures under acidic or basic catalysis, followed by aging, drying and calcination. MBGNs are further obtained by introducing structure-directing agents such as cetyltrimethylammonium bromide (CTAB) or Pluronic block copolymers, which act as templates for the ordered mesoporous structure. After the gelation and condensation steps, the template is removed through calcination or solvent extraction, yielding nanoparticles with high surface area, tunable pore sizes, and well-defined mesostructures. This mesoporosity provides additional drug-loading capacity and enhances ion release profiles compared to conventional BGNs [[80], [81], [82], [83]].

In addition to this intrinsic mesoporosity, several strategies have been developed to further improve the interaction between drug molecules and nanoparticle carriers, improving both loading efficiency and release control. Surface modification and functionalization represent the most used approaches to tailor the physicochemical properties of the carrier toward specific therapeutic molecules. The incorporation of reactive groups such as amine (-NH_2_), carboxyl (-COOH) or thiol (-SH) through silane coupling or polymer grafting enhances electrostatic and hydrogen-bonding interactions, thereby increasing drug adsorption and retention within the mesoporous structure. These modifications can also be designed to introduce temperature-, pH-, or redox-sensitive linkages that enable stimuli-responsive release, allowing environmental changes to trigger controlled drug release. In addition, coating the nanoparticles with organic molecules, such as PDA, provides catechol and amine moieties capable of forming covalent or π–π stacking interactions with aromatic drug molecules, combining strong adhesion with redox responsiveness [[84], [85], [86]].

For instance, Jiang et al. [87] demonstrated that amino-functionalization of MBGNs with 3-(aminopropyl)triethoxysilane (APTES) increased the loading rate of gentamicin sulfate from 48.90 % to 62.92 %. Ilyas et al. [88] showed that amination not only drastically increased the loading capacity of *Boswellia sacra* extract (from 1.9 mg to 9.9 mg), but also significantly prolonged its release, taking 168 h for controlled release compared to almost complete release (96.6 %) in just 24 h for unmodified particles. Yao et al. [89] reported that modifying the surface of MBGNs with alginate improved the loading efficiency of simvastatin, increasing it from 24.5 % to 31.2 %, while simultaneously delaying drug release due to the hindrance caused by the alginate polymer chains on the surface. A study by Damian-Buda et al. [90] demonstrated that amination was essential for the loading of clove oil, resulting in a controlled release profile over 14 days that was attributed to the electrostatic bonding between the drug and the protonated amino groups on the surface. In the work of Tabia et al. [91], the same surface modification with APTES was used to overcome the negative surface of MBGNs, allowing the loading of amoxicillin (a negative drug). Furthermore, the modification altered the release kinetics, which proved to be faster as the Mg^2+^ content (and therefore the surface area and the number of grafted amino groups) increased.

Alternative synthesis methods including spray pyrolysis, flame synthesis, hydrothermal methods, and emulsion-assisted sol–gel have also been investigated. These techniques allow fine-tuning of particle size (typically 30–300 nm), morphology (solid or mesoporous), and surface chemistry, characteristics whose control is crucial for the design of nanoparticles for CS and drug delivery applications [[92], [93], [94], [95]].

Recent computational approaches, including molecular dynamics and Monte Carlo simulations, have provided interesting insights into the atomic organization of silica networks and the mechanisms of ion release and network depolymerization. These models help correlate compositional parameters with glass stability and bioactivity, complementing experimental findings, particularly for predicting ion release, antibacterial effects, and regenerative potential. As noted by Khamehchi et al. [96], computational approaches provide a robust framework for assessing the stochastic nature of ion transport and the probability of effective, safe ion release. These models allow researchers to investigate how fundamental properties, such as ionic size, influence diffusion rates and bioavailability. By integrating mathematical models with Monte Carlo simulations, it is possible to account for uncertainties in experimental data and provide a probabilistic assessment of performance, which is essential for designing advanced biomaterials with controlled and predictable therapeutic delivery.

For example, Khamehchi et al. [96] successfully modeled the concentration of Ag^+^ and Zn^2+^ ions from BGs, finding that the models accurately estimated the release behavior. Results revealed that the smaller ionic size of Zn^2+^ facilitates more rapid diffusion compared to Ag^+^, which is released more slowly. The simulation predicted sustained therapeutic release for Ag^+^ for approximately 9 days, while Zn^2+^ showed a rapid initial release that decreased within 5 days, offering insights for optimizing materials for antibacterial protection or early-stage regeneration. In a related application, Mahmoudi Alashti et al. [97] used Tafel equation modeling and Monte Carlo simulation to analyze the corrosion behavior of a biomedical Ti alloy. Their model reliably predicted current densities and provided a probabilistic evaluation that accounted for parameter uncertainties, demonstrating the utility of modeling in verifying the durability and reliability of biomaterials in physiological environments.

## 3 Inorganic shells

**Fig. 2:**
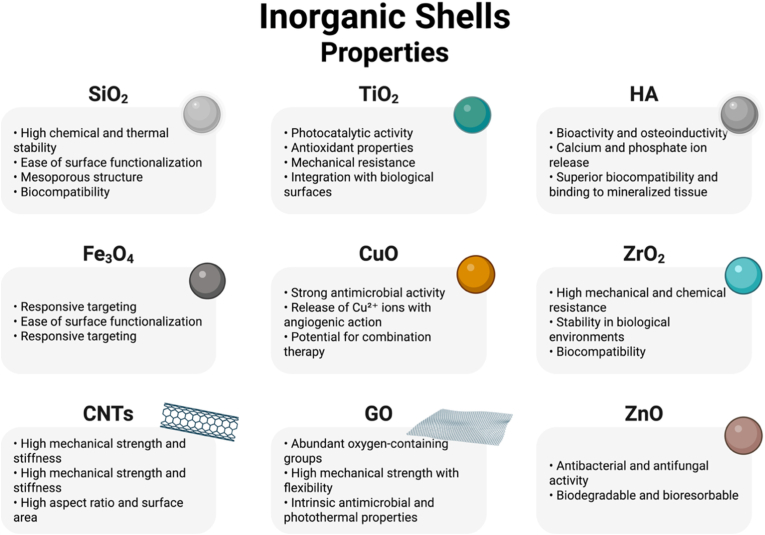
Main inorganic shells and their properties (HA – hydroxyapatite, CNTs – carbon nanotubes, GO – graphene oxide). Created in BioRender. Gabriel, A. (2025) https://BioRender.com/jl3i4qx.

The use of inorganic materials in the formulation of CS nanostructures has been widely explored lately due to their intrinsic physicochemical properties of robustness, tunable surface functionalities, and often superior compatibility with harsh physiological conditions or high-temperature processes. For biomedical applications, the choice of an inorganic shell is usually driven by the need for chemical stability, negligible or low degradation rates, and the possibility of conferring additional functionalities, such as catalytic, magnetic, optical, or antimicrobial behavior [35,[98], [99], [100]]. Common examples of inorganic shell materials include silica (SiO_2_), titanium dioxide (TiO_2_), HA (Ca_10_(PO_4_)_6_(OH)_2_), other Ca-P, iron oxide (Fe_3_O_4_/Fe_2_O_3_), cerium oxide (CeO_2_), and various metal and metal oxide nanoparticles [101,102]. The use of silica as a shell-forming material is facilitated by the ease of synthesis of the material by sol-gel or Stöber process [103], exhibiting biocompatibility and high surface area. In addition, silica shells allow covalent surface functionalization through silane coupling agents, which enables the attachment of targeting ligands, polymers, or responsive moieties [35,43,[104], [105], [106]]. Silica shells can also be applied as partial coatings on cells, providing mechanical support that enhances survival and preserves functionality in suspension [107]. While silica possesses poor biological activity, the use of HA and other Ca-P shells can ensure additional osteoconductivity and chemical similarity to the bone structure [[108], [109], [110]]. On the other hand, TiO_2_ shells could offer photoresponsive behavior under UV or visible radiation, with applications in photodynamic or antimicrobial therapy, especially when these systems contain photosensitizers [[111], [112], [113]]. Iron oxide and cerium oxide shells exhibit magnetic and redox properties, respectively. Magnetic iron oxide shells can be used for magnetic targeting [32,35,114], MRI contrast or hyperthermia therapy [19,114], while CeO_2_ shells exhibit antioxidant properties by scavenging reactive oxygen species (ROS) through reversible redox cycling between Ce^3+^ and Ce^4+^ [19]. Fig. 2 summarizes the main inorganic materials and the advantages of their use. These examples show the multifunctional features that make inorganic shells attractive not only for tissue engineering or drug delivery, but also for applications in bioimaging, cancer therapy and biosensing.

Despite their promising properties, one of the most critical limitations of many conventional inorganic shells is their reduced biological activity or bioresorbability [115,116]. However, this drawback can be addressed through engineering the shell to be porous, thin or degradable, thereby enabling the diffusion of biological fluids to the core. This reinforces the rationale for using BGN cores, which can offer ion release and tissue regeneration functionalities that inert shells cannot offer [117,118]. Thus, the strategic combination of BGN cores with an engineered inorganic functional shell can yield hybrid nanocarriers that are structurally robust, biologically functional, and responsive to complex physiological environments [19,104].

### 3.1 Zirconia-based shells

**Fig. 3:**
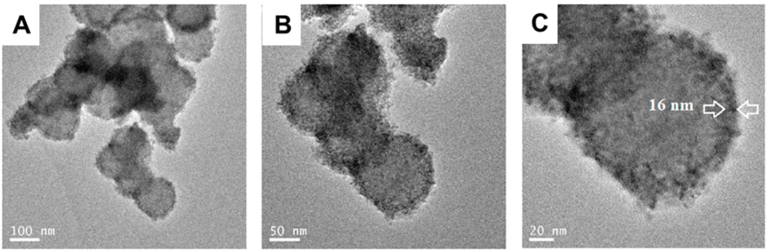
High resolution transmission electron microscopy (HR-TEM) images of the CS BGNs@ZrO_2_ nanoparticles at (A-C) different magnifications. Reprinted and adapted with permission from Ref. [119]. Copyright 2021 Elsevier.

In a study conducted by Hoomehr et al. [119], BGNs (70 % SiO_2_ – 30 % CaO) (mol) were synthesized using sol-gel methodology in ethanolic solution using TEOS as silica precursor, Ca(NO_3_)_2_•4H_2_O as calcium precursor, and NH_4_OH as catalyst, under stirring for 4 h. The material was then calcined at 600 °C for 3 h. These BGNs served as cores for the formation of CS structures with zirconia (BGN@ZrO_2_) shells. The shell formation was achieved through a sol–gel process, in which zirconia precursor (zirconium (IV) propoxide) in ethanol was left to adsorb onto the BGN core surfaces for 4 h, followed by hydrolysis and calcination at 600 °C for 3 h to crystallize the ZrO_2_ shell. The obtained results showed that this method ensured the formation of a uniform and coherent shell layer, with particle diameter of approximately 118 ± 9 nm for BGNs, and 145 ± 8 nm for CS NP, corresponding to a zirconia shell thickness of 16 ± 4 nm, as shown in Fig. 3. The resulting BGN@ZrO_2_ CS nanoparticles were deposited onto biomedical grade Ti_6_Al_4_V substrates using electrophoretic deposition (EPD), aiming for a uniform coating thickness and strong adhesion. The bioactivity of the nanoparticles was tested by conducting immersion tests in simulated body fluid (SBF) for 7 days. The results showed that, for both materials, Ca-P deposits were formed. However, the Ca-P layer was reduced for the CS particles due to the presence of the zirconia shell, which acted as a diffusion barrier that limited the ion release from the BGN core, thus directly affecting the HA formation. The metal substrate coated with CS nanoparticles presented a more coherent surface with fewer cracks compared to the uncoated substrate. These improvements were attributed to the zirconia shell, which conferred a rougher surface to the material. This further led to a higher interlocking and adhesion with the substrate, in addition to reducing the voltage required to perform the deposition and obtain a homogeneous coating [119].

**Fig. 4:**
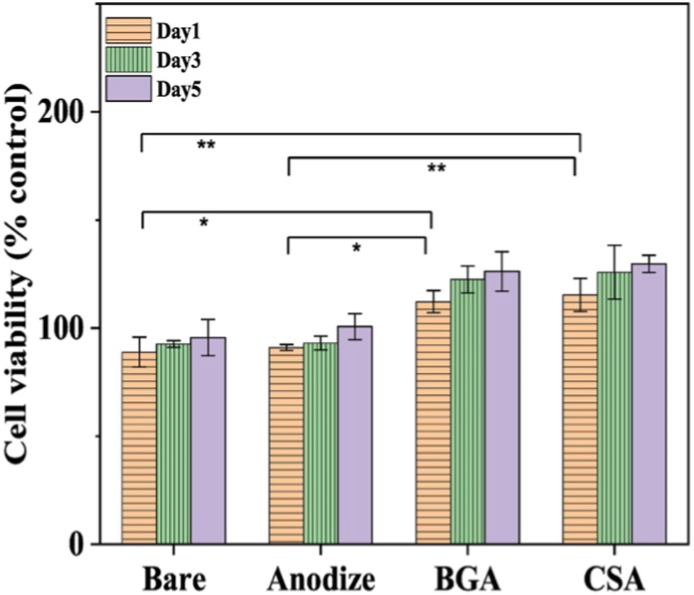
MG-63 cell viability on bare, anodized, BG (BGA) and CS BG (CSA) coated Ti6Al4V substrates after 1, 3 and 5 days. Reprinted with permission from Ref. [116]. Copyright 2022 Elsevier.

Further investigations led by the same authors showed that the same CS materials could be successfully used to coat anodically oxidized Ti_6_Al_4_V surfaces through cathodic EPD. The particles were treated with dopamine and polyethyleneimine to enhance adhesion and suspended in ethanol, followed by performing EPD under the previously optimized parameters. The obtained materials (bare, anodized, and anodized substrates coated with BG and CS particles) were characterized in terms of their barrier performance, bioactivity, and cell viability. The CS material presented the highest corrosion resistance, when compared with the simple BGN coating. This improvement was attributed to the superior mechanical integrity of the zirconia shell, that ensured a better cohesion between the particles, thus reducing the thickness of the coating and minimizing the formation of microcracks. The substrate coated with CS nanoparticles maintained its corrosion barrier function over 28 days of immersion in SBF, while the material coated with BGNs exhibited high porosity and crack propagation. The authors concluded that the zirconia shell altered the formation kinetics of Ca-P deposits by acting as a barrier that limited the release of ions from the core. After 28 days of immersion, both materials showed the formation of dense deposits of Ca-P and crystalline HA. However, the control over the core dissolution served as a tool for controlling pH variation, providing a more stable environment for cell activity. In terms of cell viability, both coatings (BG and CS) supported MG-63 osteoblast-like cell proliferation, showing similar proliferation rate at all tested time points. For instance, after 5 days, the cell viability on the CS coating was 129 ± 4 % (relative to control), a value similar to that observed on the simple BG coating (shown in Fig. 4). However, the key biological advantage of the CS coating was its superior support for cell attachment and spreading. The authors attributed this effect to the excellent pH control provided by the zirconia shell, which prevented the rapid pH increase observed for the simple BG group. This pH stability supports a more favorable microenvironment for cell metabolism and morphology. Thus, the results suggest that the use of zirconia as a shell and this material as a coating in orthopedic and dental implants may present suitable biofunctionality and corrosion resistance [116].

### 3.2 Copper oxide-based shells

**Fig. 5:**
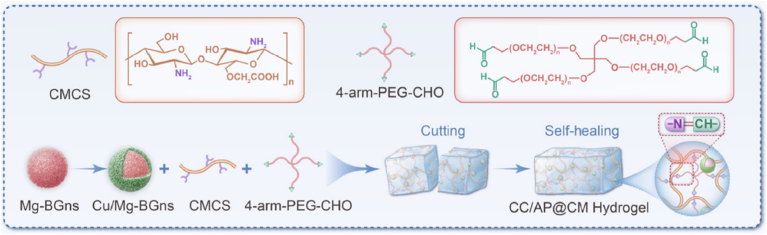
Schematic diagram illustrating the synthesis process of the hydrogel incorporating Mg-Cu/Mg CS particles (CC/AP@CM). (CMCS - carboxymethyl chitosan, PEG-CHO - 4-arm polyethylene glycol aldehyde, Cu/Mg-BGns - Cu-coated Mg-doped bioactive glass nanoparticles). Reprinted and adapted with permission from Ref. [120]. Copyright 2025 Elsevier.

Another promising CS material based on BGNs was produced by Lyu et al. [120] using BGN (65 % SiO_2_ – 25 % CaO – 10 % MgO) (mol) core and a copper-rich porous shell. The core particles were synthesized in a sol-gel process in ethanolic solution using TEOS as silica precursor, Ca(NO_3_)_2_•4H_2_O as calcium precursor, Mg(NO_3_)_2_ as magnesium source and NH_4_OH as catalyst, under stirring for 2 h. The material was calcined at 750 °C for 2 h. To produce the CS particles, the cores were dispersed in water and combined with NH_4_OH, CTAB, ethanol, TEOS and Cu/ascorbic acid complex as copper source, left to incorporate for 24 h, followed by drying and calcination at 750 °C for 2 h. These nanoparticles were incorporated into an injectable, self-healing and adhesive hydrogel matrix, synthesized via Schiff base crosslinking of 4-arm polyethylene glycol aldehyde (PEG-CHO) and carboxymethyl chitosan (CMCS), aimed at treating infectious wounds through stage-specific immunomodulation, as represented in Fig. 5. The BGN core was engineered to release magnesium (Mg^2+^) ions, known for their role in promoting tissue regeneration and anti-inflammatory responses. The shell was designed to release copper (Cu^2+^) ions, which possess antimicrobial properties and can stimulate angiogenesis. In this study, the copper-rich shell played an important functional role.

**Fig. 6:**
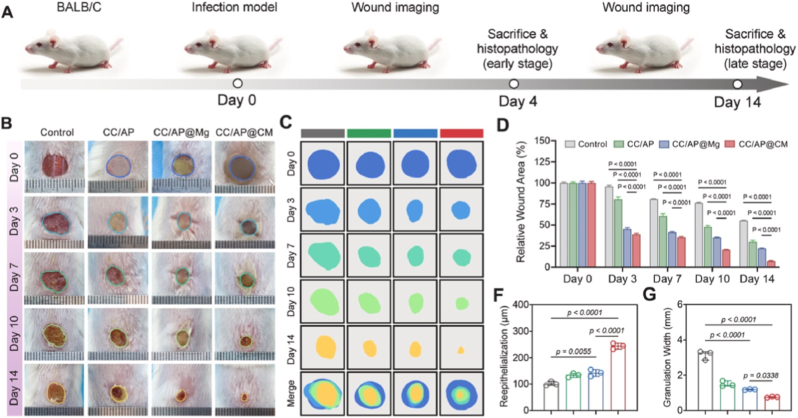
*In vivo* assessment of the different types of materials; carboxymethyl chitosan (CC/AP), carboxymethyl chitosan loaded with MgO-BGNs (CC/AP@Mg), and carboxymethyl chitosan loaded with CS Cu@MgO-BGNs (CC/AP@CM). (A) Schematic illustration of mouse infection creation and experiments timeline, (B–C) representative photos of infectious wounds at different time points, (D) area of the unhealed wounds at different intervals for each group, (F) thickness of re-epithelialization of the wound on day 14, (G) granulation width of re-epithelialization of the wound on day 14. Reprinted with permission from Ref. [120]. Copyright 2025 Elsevier.

More precisely, the high level of Cu^2+^ ion release in the first 3 days induced macrophage polarization towards the pro-inflammatory M1 phenotype and enhanced the expression of pro-inflammatory cytokines. It also exerted strong antibacterial effects against methicillin-resistant *Staphylococcus aureus*, as evidenced by ROS generation, membrane disruption, and lipid peroxidation in bacterial cells. These results are in line with the early inflammatory stages of wound healing. After 5 days of testing, the shell degraded, delaying the initial Mg^2+^ ion release. This change mediated the phenotypic switch of macrophages from the M1 to the M2 phenotype, characterized by increased expression of anti-inflammatory markers and reduced pro-inflammatory signals. This second phase promoted fibroblast proliferation, triggered angiogenesis, and collagen deposition, leading to accelerated wound closure and improved tissue remodeling. This temporal separation of the release of different ions enabled a strategic biphasic immune modulation, capable of controlling prolonged inflammation, reduced infection burden early, and fostered regenerative outcomes in the later phase. Compared to the materials without the copper shell, the BGNs-based CS particles showed a compromise in antibacterial performance and delayed macrophage activation, while the nanoparticles without Mg^2+^ failed to promote the transition from inflammation to tissue regeneration. These results were further correlated with *in vivo* studies, performed on a mouse full-thickness defect model. As illustrated in Fig. 6, dorsal wounds were created and exposed to a single treatment of phosphate buffer saline solution (PBS, control), carboxymethyl chitosan (CC/AP), carboxymethyl chitosan loaded with MgO-BGNs (CC/AP@Mg), or carboxymethyl chitosan loaded with CS Cu@MgO-BGNs (CC/AP@CM) hydrogels. The healing progress was monitored over 14 days and the results demonstrated that the CC/AP@CM hydrogel significantly accelerated wound closure. Moreover, this group showed no signs of infection or pus, which was present for the other groups. On Day 14, the CC/AP@CM group exhibited a continuous and coherent epidermis and significantly increased re-epithelialization thickness. This group also showed superior wound contraction, as measured by a reduced granulation tissue width. The authors concluded that this CS system acts as a functional trigger for the early immune defense phase, while enabling a transition to regenerative signaling through protected core-delivered ions. Furthermore, its incorporation into matrices that can modulate the controlled release of these nanomaterials or even present antibacterial efficacy demonstrate great potential for the treatment of infectious wounds [120].

**Fig. 7:**
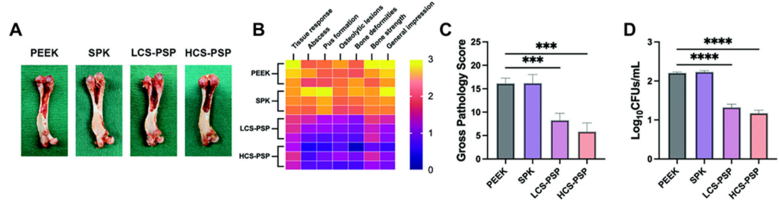
In vivo results of implant-related osteomyelitis model treated with different types of materials: simple PEEK, sulfonated PEEK (SPK), sulfonated PEEK coated with low concentration of PDA-coated CS Cu-Sr BGNs (LCS-PSP) and sulfonated PEEK coated with higher concentration of PDA-coated CS Cu-Sr BGNs (HCS-PSP). (A) Gross photos of the femurs after implant removal, (B,C) scoring of the gross bone pathology of the implanted femurs, (D) quantitative analysis of the bacteria surrounding bone tissue after treatment with the materials. Reprinted and adapted with permission from Ref. [121]. Copyright 2023 Wiley.

Wu et al. [121] developed a bilayer CS system consisting of Cu/Sr-doped BGNs (70 % SiO_2_ – 20 % CaO – 10 % SrO) (mol) as core, and CuO shell. Strontium-doped BGNs were synthesized via a sol-gel process in ethanol using TEOS as silica precursor, Ca(NO_3_)_2_•4H_2_O as calcium precursor, Sr(NO_3_)_2_ as strontium source and NH_4_OH as catalyst, under stirring for 2 h. The material was calcined at 750 °C for 4 h. The particles were dispersed in water and mixed with NH_4_OH, CTAB, ethanol, TEOS and Cu/ascorbic acid complex as copper source. The mixture was left to incorporate for 24h, dried and calcined at 750 °C for 3 h, resulting in Cu-Sr bilayer BGNs. The nanoparticles were attached to sulfonated polyetheretherketone (PEEK) by first coating the nanoparticles with PDA to create a multifunctional system for antibacterial therapy and tissue regeneration. In vitro assays demonstrated sustained release of Sr^2+^ and Cu^2+^ ions, leading to enhanced osteogenic differentiation of MC3T3-E1 cells, as evidenced by increased viability, alkaline phosphatase (ALP) activity, and upregulation of osteogenic markers (Runx2, osteocalcin - OCN). Antibacterial assays confirmed strong inhibition against *E. coli* and *S. aureus*. Immunological studies showed that CuO shell promoted a favorable immunomodulatory response, shifting macrophages toward the pro-regenerative M2 phenotype and increasing anti-inflammatory cytokine expression, thereby supporting a microenvironment conducive to bone healing. *In vivo* assays in a rat critical size calvarial defect model showed that these nanoparticles induced robust and dense new bone formation compared to bare BGNs or controls, while also preventing bacterial colonization, as shown in Fig. 7. Histological analyzes confirmed minimal inflammatory response, highlighting both the osteogenic and immunoregulatory capacity of the system.

The methodologies explored by Lyu et al. [120] and Wu et al. [121] to manufacture copper-containing shells are similar, relying on a complex and energy-intensive two-step sol-gel and calcination process. Both studies first synthesize the therapeutic ion-doped core (Mg-BGN or Sr-BGN) via sol-gel. This core is then used as a seed for a second sol-gel reaction, where a silica network shell containing a Cu/ascorbic acid complex is deposited, followed by a second calcination. While this dual-calcination approach is laborious and energetically costly, it was shown to be highly effective for its intended purpose. The high-temperature processing successfully fused the layers into a distinct, durable bilayer glass structure. This structural success is directly reflected in the ion release results: both studies achieved their goal of spatiotemporal, sequential release, where the outer copper layer dissolved rapidly in the early stages, followed by the sustained release of the inner core's ion (Mg^2+^ or Sr^2+^) in the later stages. Therefore, the methodological complexity was a necessary trade-off to create a purely inorganic, staged-degradation system capable of sequential ion release, which a simpler, low-temperature polymer coating would not have achieved.

### 3.3 Functional ceramic-based shells

**Fig. 8:**
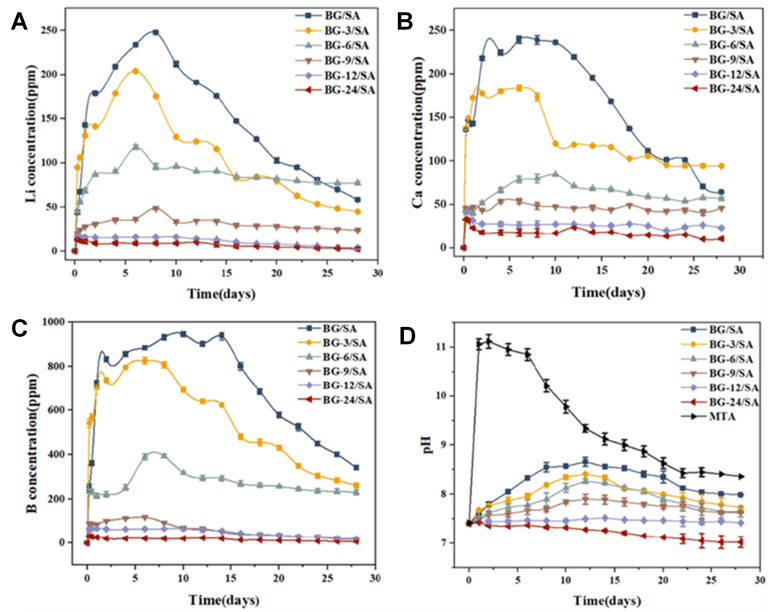
Ion dissolution during mineralization of CS HA@BG nanoparticles incorporated at different concentration in a sodium alginate matrix (BG-x/SA). (A) Li^+^, (B) Ca^2+^, (C) B^3+^ and (D) pH trend. Reprinted and adapted with permission from Ref. [122]. Copyright 2025 IOPSCIENCE.

Further expanding the applications of CS BGN-based systems, a sodium alginate (SA)-based nanocomposite cement containing different concentrations of BGN CS nanoparticles (BGN-x/SA, where x denotes the amount of BGNs) was developed by Xu et al. [122] for direct pulp capping applications. This composite cement was composed of borate-based BGNs (80 % B_2_O_3_ – 10 % CaO – 10 % Li_2_O) (mol) as the core and an in situ-formed mesoporous HA shell (BGN@HA), dispersed in SA. The BGN cores were synthesized by melt-quenching the precursors at 1100 °C for 25 min, crushing, sieving and converting them to spherical shapes by flame spraying. These cores were immersed in K_2_HPO_4_ solution for different time points and calcined at 750 °C for 2 h to obtain the CS materials. This variation did not cause a significant change in the overall size of the microspheres. However, it directly affected the surface morphology: as the reaction time increased, the initially smooth glass surface became rougher and transformed into a HA shell. Longer reaction times also led to a more complete transformation and higher crystallinity of the HA shell, and the internal glassy core began to transform into looser particles. The results obtained showed that the HA shell conferred beneficial properties to the material and the resulting cement formulation. Specifically, the HA shell contributed to the improved mechanical properties, demonstrating superior compressive strength, reduced setting time, and enhanced anti-washout ability. It also enabled tunable ion release and pH control, as the HA shell modulated the release kinetics of therapeutic ions (Li^+^, Ca^2+^, and B^3+^) from the BGN core, as depicted in Fig. 8, further delaying the ion release and producing a moderate alkaline microenvironment, favorable for pulp healing. Moreover, the shell served as a nucleation platform for apatite crystallization, significantly enhancing the material's bioactivity and apatite formation. From a biological point of view, the BGN@HA containing cement exhibited improved cytocompatibility with human dental pulp cells (hDPCs), supporting proliferation and spreading, while enhancing odontogenic differentiation. Simultaneously, the presence of the shell did not impair, but in fact supported the antibacterial efficacy of the composite cement, showing strong inhibitory effects against *E. coli, S. aureus,* and *S. mutans* [122].

**Fig. 9:**
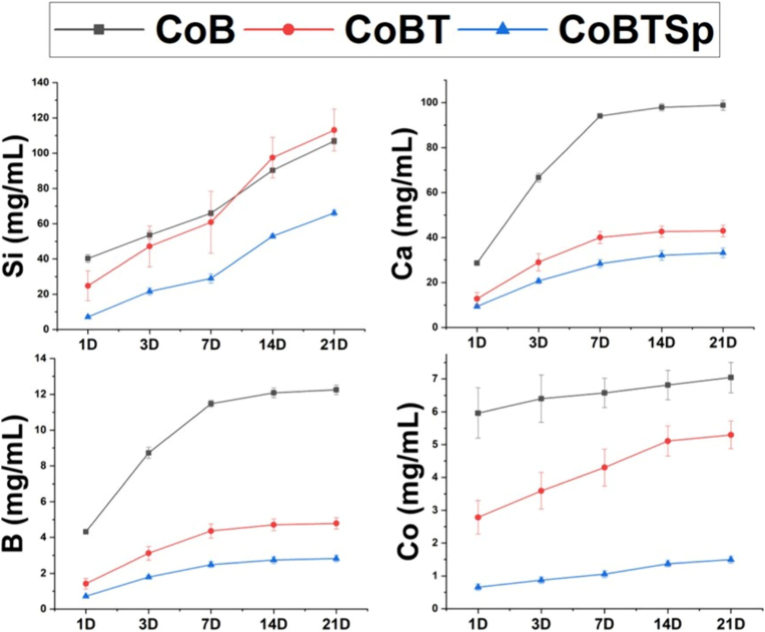
Ion release after the materials (Co-doped MBGNs (CoB), CS silica-coated Co-doped (CoBT) and CS silica-SPIONs-coated Co-doped MBGN (CoBTSp)) were immersed in Tris buffer for 1, 3, 7, 14, and 21 days. Reprinted with permission from Ref. [123]. Copyright 2025 American Chemical Society.

Vitázková et al. [123] developed CS nanoparticles with a MBGN core (55 % SiO_2_ – 30 % CaO – 10 % CoO – 5 % B_2_O_3_) (mol) and a silica shell functionalized with superparamagnetic iron oxide nanoparticles (SPIONs). The MBGNs were prepared using microemulsion-assisted sol-gel method in water using CTAB, ethyl acetate, NH_4_OH, TEOS, Ca(NO_3_)_2_•4H_2_O, boric acid, and Co(NO_3_)_2_ as cobalt source, under stirring for 2 h. The material was calcined at 650 °C for 3 h. The silica coating was produced by dispersing the MBGNs in ethanol, NH_4_OH and TEOS, under stirring for 2 h and drying overnight at 60 °C. These nanoparticles were suspended in water alongside FeCl_3_ and FeSO_4_ under continuous stirring at room temperature, with NH_4_OH added dropwise for 10 min to induce the co-precipitation of the SPIONs, and dried overnight at 60 °C. Release assays showed that the hybrid system is capable of maintaining controlled release of Si(OH)_4_, Ca^2+^, Co^2+^, and B^3+^ ions and it is attributed to the shell the ability to prevent burst release and improve colloidal stability. Uncoated Co-doped MBGNs (CoB) exhibited the fastest degradation rate and ion release, primarily due to its higher specific surface area, which increases solid-liquid interactions. The CS silica-coated Co-MBGNs (CoBT) and silica-SPIONs-coated Co-MBGNs (COBTSp) particles showed slower release of Ca^2+^, Co^2+^, and B^3+^, confirming that the silica shell acts as an effective selective diffusion barrier. The CoBTSp demonstrated the most pronounced reduction of ion release, maintaining bioactivity while preventing the concentration-dependent cytotoxicity. Biological assays revealed excellent cytocompatibility with MG-63 osteoblast-like cells and MC3T3-E1 pre-osteoblasts, sustaining >80 % viability at concentrations up to 200 μg/mL, with no detectable genotoxicity. The nanosystem strongly enhanced angiogenesis, up-regulating vascular endothelial growth factor-A (VEGFA) (up to 31-fold), hypoxia-inducible factor 1A (HIF1A), and and fibroblast growth factor 2 (FGF2) expression, while also promoting osteogenesis, as demonstrated by increased RUNX2 and COL18A1 expression and enhanced extracellular mineralization in MC3T3-E1 cells. A reduction of TP53 expression suggested attenuation of pro-apoptotic signaling. DNA fragmentation assays confirmed the absence of detectable genotoxicity, as cells exposed to the CS nanosystem showed no apoptotic DNA laddering even at the highest tested concentrations, as shown in Fig. 9.

The shell fabrication methodologies presented by Vitázková et al. [123] and Xu et al. [122] are fundamentally different, reflecting their opposing therapeutic goals. Vitázková et al. [123] employed a conventional, low-temperature sol-gel method based on the Stöber approach, where TEOS was hydrolyzed to deposit an additive silica coating onto the existing Co-doped MBGN core. This process, which only required drying at 60 °C, was highly effective for its specific purpose: to passivate the core and create a physical diffusion barrier. The success of this simple method is evident in the results, as the SiO_2_ shell significantly attenuated the release kinetics of Si(OH)_4_, Ca^2+^, Co^2+^, and B^3+^ ions compared to the uncoated core, thereby improving biocompatibility by mitigating the cytotoxic burst release observed at higher concentrations. In sharp contrast, Xu et al. [122] utilized a complex, high-temperature in-situ transformation process. Their borate glass core was not passively coated; instead, it was actively corroded by immersion in a K_2_HPO_4_ solution, causing ions to leach out while a new Ca-P layer precipitated onto the surface, followed by a 400 °C calcination step to crystallize this new layer into HA. This energy-intensive method was critical for success, as it did not just coat the BGs, but transformed its surface into a crystalline, mesoporous HA layer. This HA shell proved to be superior to the original amorphous glass core by providing enhanced nucleation sites, which directly resulted in the final cement (BG-6/SA) demonstrating faster apatite formation, better anti-washout properties, and significantly improved promotion of hDPC differentiation compared to the control. Therefore, while Vitázková's method successfully created a stable diffusion barrier, Xu's more complex transformation method was essential for creating a highly bioactive, crystalline surface designed for rapid, cell-instructive bone regeneration.

## 4 Organic shells

### 4.1 Natural polymers

**Fig. 10:**
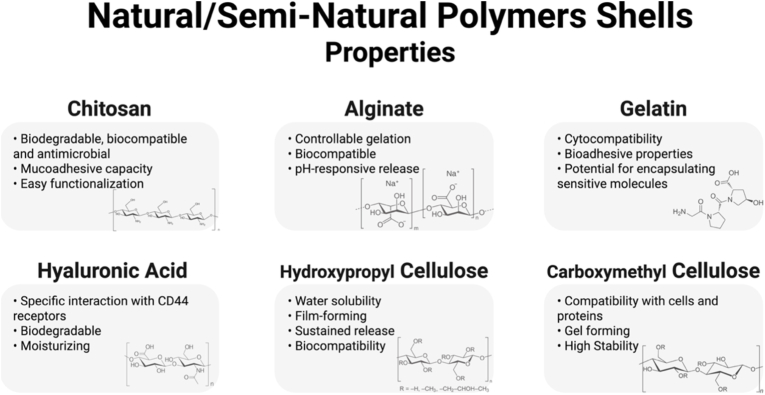
Main natural and semi-natural polymeric shells applied on BGN core and their properties. Created in BioRender. Gabriel, A. (2025) https://BioRender.com/7d1eelp.

Besides inorganic materials, different natural polymers have been extensively employed as shells in BGN-based CS nanostructures due to their outstanding biocompatibility, biodegradability and biofunctionality [100,124,125]. These polymers, shown in Fig. 10, can mimic key features of the ECM, which makes them ideal for biomedical applications such as drug delivery, tissue engineering, and regenerative medicine. Natural polymers, including chitosan, alginate, gelatin and hyaluronic acid have an abundance of functional groups (e.g. amino, carboxyl, hydroxyl), which allows and facilitates surface modifications, crosslinking and biofunctionalization [126]. Their inherent hydrophilicity and ability to form hydrogels enable the formation of a suitable microenvironment for cell adhesion, proliferation and nutrient diffusion. In this context, polymeric shells can be formed on the BGN core surface by layer-by-layer assembly, ionic gelation, electrostatic deposition, or in situ precipitation [127]. These shells can act as diffusion barriers that regulate drug release kinetics, protect therapeutic payloads from enzymatic degradation, and modulate interactions with the surrounding biological tissues [128,129]. Furthermore, many of these polymers are stimuli-responsive, allowing pH-, temperature- or ionic-strength-dependent release in pathological environments like infection sites, tumors or inflamed tissues [130].

The versatility of using these natural polymers goes beyond simple encapsulation. Their chemical composition can confer antimicrobial, hemostatic, and immunomodulatory properties that can synergize with the therapeutic potential of the BG core [131]. In the class of natural polymers, we can mention I) chitosan, which has inherent antibacterial activity and mucoadhesion properties, II) alginate, which forms ionically crosslinked hydrogels in the presence of divalent cations such as Ca^2+^ or Ba^2+^, resulting in mechanically stable matrices, III) gelatin, a denatured form of collagen, that supports cell adhesion and matrix remodeling, while hyaluronic acid plays critical roles in cell signaling and ECM hydration [125,132].

Chitosan is a polysaccharide derived from the deacetylation of chitin and has been widely used as a shell due to its antimicrobial, film-forming and ionic interaction properties. The primary amino groups allow electrostatic binding to negatively charged surfaces, such as silicate-based BGNs, as well as crosslinking with anionic agents (e.g., tripolyphosphate, alginate) [133]. Chitosan-based shells can exhibit pH-responsive swelling and degradation, with accelerated degradation under acidic conditions, making them especially suitable for tumor-targeted delivery or inflammation associated with infections [134,135]. These hybrid CS systems have been proposed for different applications, ranging from bone defect repair to transdermal delivery platforms [136,137].

#### 4.1.1 Chitosan-based shells

**Fig. 11:**
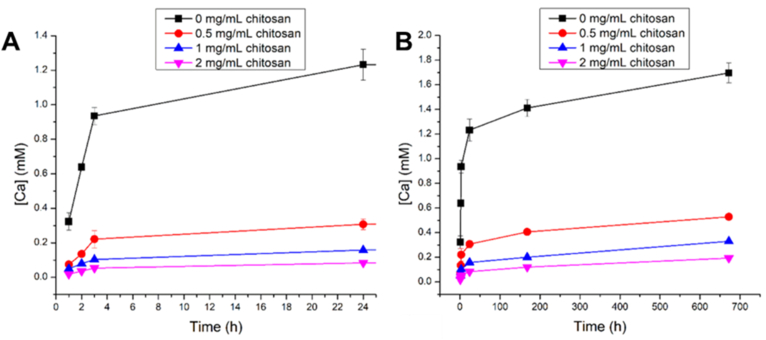
Calcium release up to (A) 24 h and (B) 672 h from bare BGNs and from chitosan-coated BGNs synthesized at different concentrations of chitosan. Reprinted and adapted from Ref. [138].under a Creative Commons CC BY License.

Uskokovic et al. [138] developed a CS structure using BGNs (40 % SiO_2_ – 24.5 % Na_2_O – 30 % CaO – 5.5 % P_2_O_5_) (mol) as the core and chitosan as shell. The core material was prepared via a sol-gel process in ethanol using TEOS, Ca(NO_3_)_2_•4H_2_O, NaNO_3_, phosphorous pentoxide and NH_4_OH, under stirring for 3 h. The material was calcined at 530 °C. The particles were suspended in water and different volumes of chitosan solution (30 mM in HCl) were added and stirred for 1 h and the product was dried in vacuum oven at 60 °C. The variation in chitosan concentration directly impacted the particle size: a higher concentration of chitosan in the solution resulted in a thicker coating, which led to a directly proportional increase in the hydrodynamic diameter of the nanoparticles. Morphologically, the coating also caused the originally discrete and non-agglomerated nanoparticles to appear embedded within an irregular polymeric matrix. These BGN@chitosan nanoparticles were aimed to modulate and control the release kinetics of Ca^2+^ ions, with potential for tailoring the bioactivity and mineralization. Key findings of this work showed that the chitosan shells acted as a diffusion barrier, substantially affecting the release profile of Ca^2+^ compared to BGNs, as shown in Fig. 11. The rate and extent of Ca^2+^ release decreased significantly with increasing chitosan shell thickness, indicating the role of the shell in tuning ion release kinetics. The Ca^2+^ release followed a non-Fickian, anomalous diffusion model, which became more pronounced with the amount of chitosan deposition. The release mechanism suggested that the release transitioned from erosion-driven to being primarily governed by matrix diffusion and polymer erosion. Furthermore, it was found that, while the presence of the chitosan shell altered the release mechanism, further increases in shell thickness did not significantly change the type of diffusion, indicating that the existence of the shell, rather than its thickness, alters/governs the release kinetics [138].

**Fig. 12:**
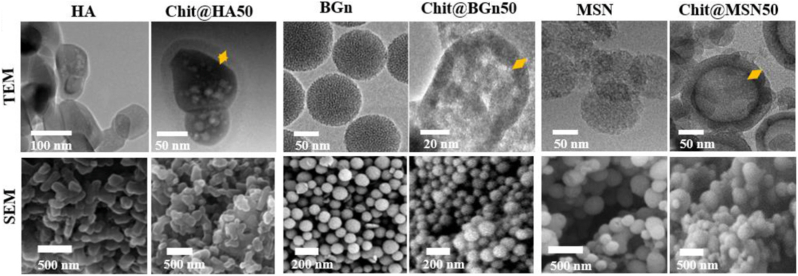
TEM and scanning electron microscopy (SEM) images of the simple HA, BGNs and MSNs and CS chitosan-based counterparts (Chit@HA50, Chit@BGn, Chit@MSNs50). Reprinted with permission from Ref. [44]. Copyright 2021 Elsevier.

In a similar study conducted by Kim et al. [44], CS nanohybrids were fabricated using bioactive inorganic nanoparticles, namely HA, BGNs (85 % SiO_2_ – 15 % CaO) (mol) or MSNs as the core, with chitosan serving as the shell, assembled via a self-assembly process in mild aqueous conditions. Fig. 12 shows micrographs of the obtained materials. Among these systems, the results showed that BGN nanohybrids exhibited distinct advantages over their HA and MSN counterparts in several key areas. More precisely, the BGN@chitosan hybrids demonstrated significantly higher surface roughness, which promoted mesenchymal stem cell adhesion and proliferation. This effect was attributed to both nano-roughened topography and ionic dissolution behavior of the BGN. Furthermore, these nanohybrids exhibited superior bone regenerative capacity in vivo, as proven by the osteogenic markers that suggested superior biological integration and regenerative efficiency ensured by the BGNs-core material in comparison to pure HA, BGNs and MSNs. In addition, the Ca^2+^ and Si(OH)_4_ ion release-mediated therapeutic benefits of the BGN core contributed to the pro-angiogenic and osteoinductive biological effects, which were absent or minimal in HA and MSN-based hybrids. Moreover, BGN-based CS nanostructure exhibited superior mechanical properties and surgical handling, maintaining an elastic behavior, with tensile strength of up to 3.2 MPa and elongation at break of approximately 400 %. Finally, the BGNs-chitosan platform enabled the controlled, multi-phase drug delivery: dexamethasone (anti-inflammatory drug) was released in the early stages, the pro-angiogenic FGF2 in the intermediate phase, and osteoinductive phenamil in the late stages, in agreement with the biological stages of bone healing [44].

The chitosan shell fabrication methods employed by Kim et al. [44] and Uskoković et al. [138] are similar in their chemical principle, both relying on the pH-dependent solubility of chitosan; yet they are applied to achieve divergent functional outcomes. Both studies utilize a low-temperature, pH-driven precipitation: chitosan is dissolved in an acidic solution, mixed with the inorganic nanoparticles, and then neutralized to trigger chitosan's insolubility and its subsequent deposition onto the core. Kim et al. [44] frame this as a sophisticated "self-assembly" driven by hydrogen bonds, while Uskoković et al. [138] describe it as a straightforward precipitation coating. Both methodologies were successful, but this was measured by different criteria. For Kim et al. [44], the chitosan shell worked as a "bioadhesive" to bind the inorganic cores, creating a new, flexible, and highly resilient hybrid material with ultrahigh inorganic content. This shell also successfully served as a distinct "compartment" for loading secondary therapeutic molecules, enabling sequential delivery. In contrast, Uskoković et al. [138] used the chitosan shell as a passive diffusion barrier. Their method worked well to prevent the undesirable burst release of Ca^2+^ ions from the BG core. This effect was proven to be "tunable," as thicker coatings predictably and effectively reduced the ion release rate. Morphologically, Kim's process appears to yield better-defined, discrete CS nanounits with a uniform shell, whereas Uskoković's method resulted in less defined structures, described as both "irregular islands" and discrete coated units.

### 4.2 Semi-natural polymers

Semi-natural polymers, derived from structural modifications of natural polymers, allow the formation of materials with new characteristics. In the class of semi-natural polymers, as shown in Fig. 8, we can mention hydroxypropyl cellulose (HPC) or carboxymethyl cellulose (CMC), which combine the bioactivity of natural materials with improved solubility, stability, and processability. These polymers can serve as hydrophilic, flexible shell layers that respond to temperature or solvent polarity, thus facilitating smart drug delivery behavior *in vivo* [139,140].

#### 4.2.1 HPC-based shells

In order to create a smart CS nanoparticle for bone cancer therapy, Arabyazdi et al. [141] developed a material consisting of a magnetic-modified BGN (40 % SiO_2_ – 10 % Fe_2_O_3_ – 10 % Li_2_O – 36 % CaO – 4 % P_2_O_5_) (mol) core and a thermo-responsive HPC shell, enabling temperature-triggered release of doxorubicin. The core material was prepared following a sol-gel process in HNO_3_ solution using TEOS, and triethyl phosphate (TEP) under stirring for 1 h, followed by the addition of Li_2_O, Ca(NO_3_)_2_ and ferrite, aged for 7 days and calcined at 700 °C for 24 h. The particles were functionalized with methacryloxypropyl trimethoxysilane in 2-propanol at 80 °C for 30 min and dried at 90 °C for 18 h. The functionalized particles were mixed with oxidized HPC under agitation for 12 h and oven dried at 40 °C for 12 h. The HPC shell was covalently grafted onto the surface, forming a stable and responsive barrier. The shell of the CS material provided precise thermal control over drug release, by remaining impermeable at physiological temperatures, and rapidly releasing the chemotherapeutic agent when exposed to mild hyperthermia, achieved via magnetic stimulation. It was shown that this shell-controlled release mechanism was able to minimize passive leakage in addition to enhancing therapeutic specificity. In addition, the shell enhanced the colloidal stability, biocompatibility, and cell adhesion properties of the system, showing promising properties as multifunctional platforms for targeted bone cancer therapy [141].

### 4.3 Synthetic polymers

**Fig. 13:**
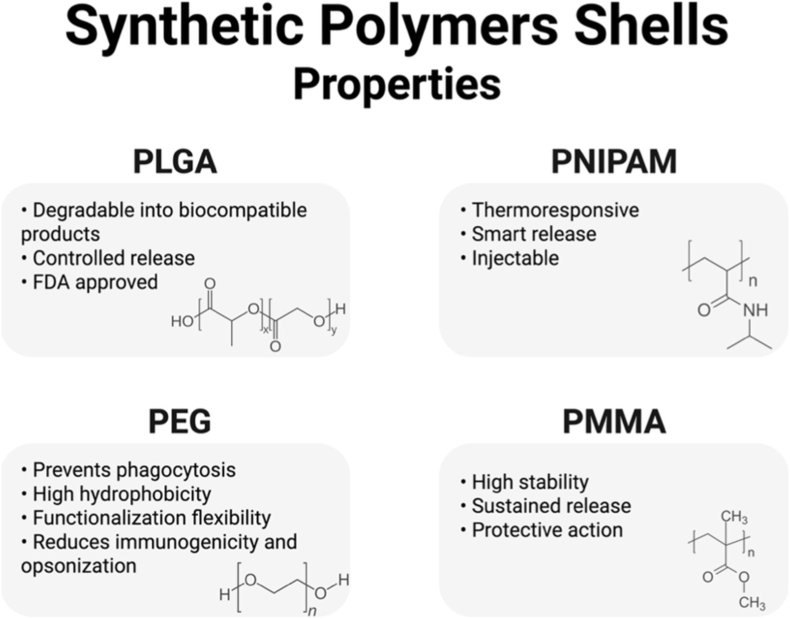
Main synthetic polymeric shells and their properties. (PLGA - poly(lactic-co-glycolic acid), PNIPAM - poly(N-isopropylacrylamide, PEG - poly(ethylene glycol), PMMA- poly(methyl methacrylate)). Created in BioRender. Gabriel, A. (2025) https://BioRender.com/2r3fbqg.

Synthetic polymers offer a higher degree of design flexibility and reproducibility than natural or semi-natural polymers, making them strong candidates for use as shells in CS nanoparticles. These materials can be engineered with precise control over their molecular weight, degradation kinetics, hydrophilicity, and functional groups [100,127,[142], [143], [144]]. In the case of drug delivery applications, synthetic polymers provide tailored release profiles, enhanced colloidal stability, and responsiveness to specific physiological triggers such as pH, temperature, or enzymatic activity, known as smart polymers [[145], [146], [147]]. In the form of nanomaterials, these polymers are used to create nanocapsules or coating layers that protect the core from premature dissolution, while allowing diffusion-controlled release of therapeutic agents or ions. Furthermore, the surface of these shells can be functionalized with targeting ligands or fluorescent tags to enhance biological specificity and in vivo performance [148,149]. The most commonly used synthetic polymers are poly(lactic-co-glycolic acid) (PLGA), poly(N-isopropylacrylamide) (PNIPAM), PEG, poly(methyl methacrylate) (PMMA), as shown in Fig. 13, and their variations and uses as copolymers and block structures.

Among these polymers, PMMA has been extensively studied due to its mechanical strength, chemical inertness, and ease of processing. When used as a shell, PMMA can modulate the kinetics of ion release while preventing rapid degradation of the core, maintaining biocompatibility in biological environments [149,150]. PNIPAM is a thermoresponsive polymer with a low critical solution temperature (LCST) around 32 °C, which confers temperature-triggered drug release properties [151]. CS structures using PNIPAM shells remain stable at room temperature and undergo conformational changes upon contact with the human body, thus promoting controlled release. This property is particularly useful in localized hyperthermia or tumor-targeting therapies. Another widely used polymer is PLGA, a biodegradable copolymer with excellent encapsulation capacity and tunable degradation rates [144,152]. Overall, the shells made out of synthetic polymers offer high adaptability and fine-tuning ability of the physicochemical and biological properties of BGN-based CS nanoparticles, which is particularly important for advancing their use in different biomedical applications.

#### 4.3.1 PMMA-based shells

**Fig. 14:**
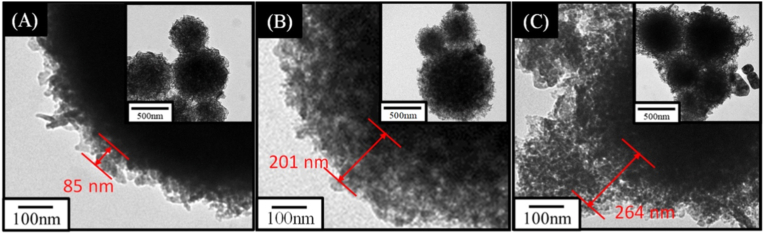
TEM images of the CS nanoparticles prepared with different methyl methacrylate (MMA): pre-MBGNs ratios: (A) 1:1, (B) 3:1, and (C) 5:1, respectively. Reprinted with permission from Ref. [153]. Copyright 2023 Elsevier.

Lin et al. [153] developed a CS composite consisting of MBGNs (80 % SiO_2_ – 15 % CaO – 5 % P_2_O_5_) (mol) coated with a PMMA shell, as shown in Fig. 14, and incorporated it into electrospun fiber scaffolds for potential tissue engineering applications. The MBGNs were synthesized by spray pyrolysis by mixing TEOS, Ca(NO_3_)_2_•4H_2_O and TEP in ethanol alongside PEG and water. The solution was taken to an ultrasonic humidifier, and the atomized droplets were introduced in a three-stage temperature furnace for calcination at 200 °C, 700 °C and 350 °C. The particles were obtained after 12 h of settling and desiccation. Then, the cores were submitted to surface modification by mixing with NaOH solution for 12 h, neutralized, washed, and dried. The particles were coated with PMMA via seed polymerization in water under stirring for 12 h, with potassium persulfate initiator added and the reaction continued at 70 °C for 4 h under continuous stirring. The authors found that increasing the monomer:MBGN ratio increased the shell thickness. They also found that the polymerization temperature affected the morphology: below the optimized temperature (70 °C) no polymerization occurred, whereas above it, it caused the formation of rough-surfaced particles and severe agglomeration. When investigating the reaction time, periods below the optimum time (4 h) resulted in the formation of irregular particles, while very long times caused the PMMA to densify, forming a rougher surface and a thinner shell. This colloidal solution was submitted to electrospinning to obtain the composite fiber scaffolds. The aim of the work was to address the mechanical limitations of MBGNs by engineering a shell that improves their processability. It was found that the PMMA shell played a crucial role in improving the mechanical and biological performance of the scaffolds. Compared to the scaffolds with MBGNs without shell, the CS structures presented almost double the tensile strength, which was attributed to improved particle dispersion and interfacial bonding between the PMMA matrix and the MBGN core. This shell also facilitated an early exposure of the MBGN core in SBF, which promoted the formation of an apatite layer and accelerated the mineralization process. The shell also improved the biocompatibility of the material, as evidenced by the higher cell viability of MG-63 osteoblast-like and L929 fibroblast cells seeded on the CS MBGN@PMMA fibers than on uncoated MBGN scaffolds, suggesting that the PMMA shell ensured a more favorable cellular interface [153].

#### 4.3.2 PNIPAM-based shells

**Fig. 15:**
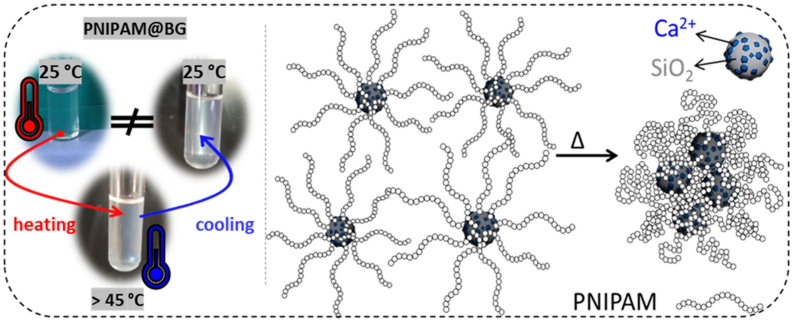
Schematic depiction and visual observation of the temperature-induced aggregation of the CS PNIPAM@BG. Reprinted and adapted with permission from Ref. [154]. Copyright 2024 Royal Society of Chemistry.

Aiming to create a stimuli-responsive nanomaterial, Nabiyan et al. [154] developed a CS system obtained by grafting a thermo-responsive polymer shell PNIPAM onto sol–gel-derived MBGNs (85 % SiO_2_ – 15 % CaO) (mol). The MBGNs were prepared via microemulsion-assisted sol-gel method in water using CTAB, ethyl acetate, NH_4_OH, TEOS, and Ca(NO_3_)_2_•4H_2_O under stirring for 4 h. The material was calcined at 700 °C for 4 h. The resulting particles were silanized with APTES in toluene at 110 °C for 24 h and the reversible addition–fragmentation chain transfer (RAFT) agent 2-(dodecylthiocarbonothioylthio)-2-methylpropionic acid (DDMAT) was attached on the surface of the particles. These particles were dispersed in dimethylformamide (DMF), mixed with N-isopropylacrylamide (NIPAM) and 4,40-azobis(4-cyanovaleric acid) (ACVA) initiator and were subjected to polymerization at 75 °C for 24 h. This system was aimed at designing a material capable of performing temperature-controlled ion delivery along with improved colloidal stability. It was found that the PNIPAM shell was responsible for modulating the behavior of the BGN core in response to temperature changes. At room temperature, the shell remained hydrated, and in a coiled state, providing steric stabilization and significantly improving the colloidal stability of BGNs in aqueous environments. When heated above the transition temperature (∼43 °C), the shell collapses into a globular conformation, resulting in a reduced stability and promoting particle aggregation, as represented in Fig. 15. This coil-to-globule transition is responsible not only for altering the scattering behavior, but also for directly affecting the ion release profile of Ca^2+^ from the BGN core. Compared to uncoated BGNs, the CS structures exhibited a reduced initial ion burst release and a more controlled ion diffusion, with temperature-dependent variations suggesting tailorable ion delivery. Overall, the PNIPAM shell conferred stimuli-responsiveness to the BGN-based material, allowing the design of a smart CS nanosystem that can release therapeutic agents in response to local temperature changes, which is particularly useful for inflammation conditions. Thus, this work highlights the potential of integrated CS architectures capable of maintaining the properties of BGNs, while offering new functionalities to the materials [154].

#### 4.3.3 Hybrid organic-based shells

**Fig. 16:**
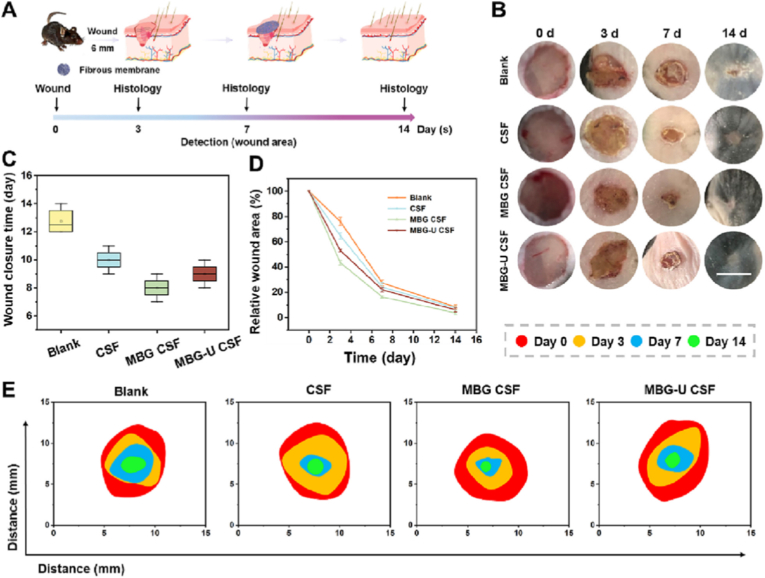
*In vivo* evaluation of wound healing of different fibers: 5-FU fibers (CSF), MBGNs CS fibers (MBG CSF) and 5-FU-loaded MBGNs CS fibers (MGB-U CSF). (A) Schematic showing the establishment of wound healing model, (B) photographs of wounds in skin during 14 days of treatment (Scale bar: 5 mm), (C) wound closure time, (D) relative wound area treated with different materials, (E) diagrammatic sketch of remaining wound area. The red, yellow, blue, and green lines indicate wound boundary at day 0, 3, 7, and 14, respectively. Reprinted with permission from Ref. [155]. Copyright 2023 Elsevier.

Similarly, a CS system for combined use in chemotherapy and wound healing for post-surgical treatment of melanoma was reported by Yuan et al. [155], where they developed a dual-functional CS electrospun nanofiber. The system was made by a core of MBGNs (60 % SiO_2_ – 36 % CaO – 4 % P_2_O_5_) (mol) in sol-gel process in water using TEOS, Ca(NO_3_)_2_4H_2_O, TEP, and HCl, under stirring, aged for 72 h and calcined at 650 °C, loaded with 5-fluorouracil (5-FU) and a PLGA, fabricating the fibers by coaxial electrospinning. The PLGA fibrous shell significantly decreased the diffusion of the drug, resulting in a linear and sustained release over 7 days. Additionally, the shell protected the MBGN core from immediate degradation and controlled water penetration, thereby avoiding premature release. Comparative analysis with fibers containing free 5-FU confirmed that the shell, in synergy with the MBGN core, was essential to minimizing burst release and maintaining therapeutic drug levels at the surgical site. In terms of biological activity, the CS structure improved both antitumor efficiency and skin regeneration compared to the uncoated material. In vitro assays using L929 cells or B16 cells showed that the material induced a significant increase in apoptosis, compared to the control and the uncoated nanoparticles. In vivo, the fibrous dressing almost completely suppressed tumor recurrence and accelerated wound closure, as shown in Fig. 16. This observation is supported by histological analyses showing increased epidermal thickness, hair follicle formation, and microvessel density. These therapeutic effects were attributed to the sustained ion release (Ca^2+^ and SiO_4_^4−^) from the MBGN core, which promoted fibroblast migration, collagen deposition, and angiogenesis [155].

**Fig. 17:**
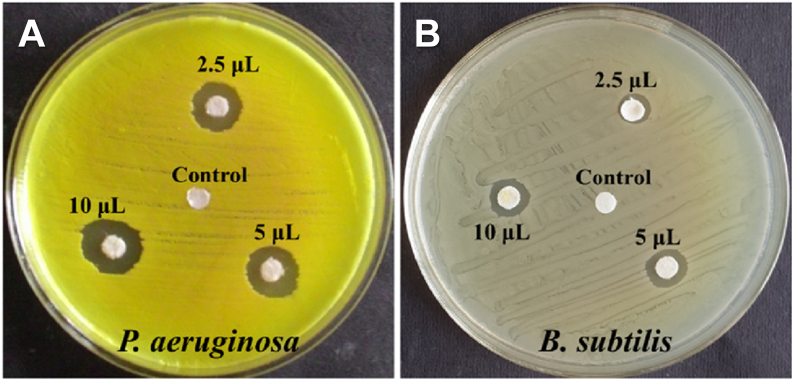
Antibacterial effect of different CS MBGN@PLGA nanoparticles against (A) *Pseudomonas aeruginosa* and (B) *Bacillus subtilis*. Reprinted and adapted with permission from Ref. [156]. Copyright 2021 Elsevier.

To obtain a pH-responsive material for chemotherapeutic delivery, Das et al. [156] developed a CS system composed of an MBGN core (80 % SiO_2_ – 15 % CaO – 5 % P_2_O_5_) (mol) with a poly-L-glutamic acid shell. The core material was prepared in sol-gel process in ethanolic solution using CTAB, TEP, Ca(NO_3_)_2_•4H_2_O, TEOS and NH_4_OH, under stirring for 6 h. The material was calcined at 650 °C for 6 h and the particles were functionalized with amine groups via silanization using APTES, heated and kept in reflux condition for 24 h. Then, the particles were conjugated to PLGA using (1-Ethyl-3-(3-dimethylaminopropyl)carbodiimide) and (N-hydroxysuccinimide) (EDC/NHS) crosslinking agents. Daunomycin (DAN) was used as a model anticancer drug, and the MBAG/PLGA nanocarriers exhibited high loading capacity (26.8 %) and pH-dependent release. After 24 h, a significantly higher release at acidic pH (77 % at pH 5.5) than at physiological pH (22 % at pH 7.4) was recorded, thus mimicking tumor microenvironments. Antibacterial assays showed that the material possesses intrinsic antimicrobial activity, with stronger inhibition against Gram-negative *Pseudomonas aeruginosa* compared to Gram-positive *Bacillus subtilis*, as shown in Fig. 17. Thus, it was demonstrated that the CS system was capable of combining controlled release with pH-responsiveness and antimicrobial activity.

**Fig. 18:**
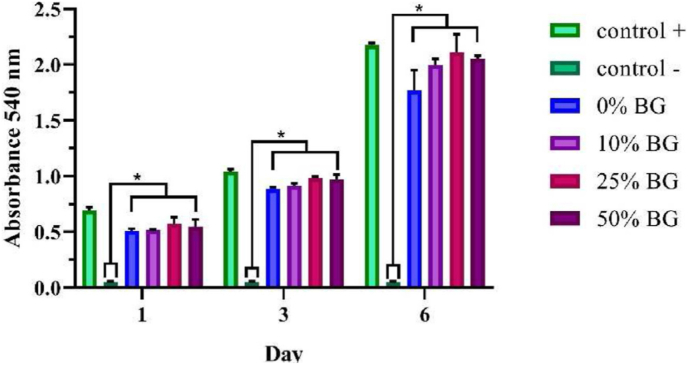
Cell viability of L929 fibroblasts seeded on CS fibers containing different concentrations of BGs. Reprinted with permission from Ref. [157]. Copyright 2022 Elsevier.

Rahmati et al. [157] fabricated CS nanofibers consisting of BGN (55 % SiO_2_ – 15 % CaO – 30 % P_2_O_5_) (mol) core and mixtures of PCL, gelatin, polyvinylidene fluoride (PVDF) and poly vinyl alcohol (PVA) for the shell, obtained via electrospinning. The core material was prepared using sol-gel methodology in water using TEP, Ca(NO_3_)_2_•4H_2_O, TEOS, and NH_4_OH, under stirring for 3 h. The material was calcined at 600 °C for 3 h. The core solution was prepared by adding the BGNs to a PCL/gelatin (70:30 v/v) in acetic and formic acid and kept stirring for 2 h. The shell solution was prepared by dissolving PVA in dimethyl sulfoxide (DMSO) and PVDF in DMF, mixed and stirred at 40 °C for 2 h. The nanofibrous membrane were prepared via coaxial electrospinning. Biological results demonstrated that the fibers maintained controlled release of bioactive ions (Si(OH)_4_, Ca^2+^), while the polymeric improved mechanical stability and prevented premature degradation. Antibacterial studies showed that the CS membranes possess antibacterial activity against *S. aureus* bacteria with the antibacterial efficiency increasing with the BG concentration. In vitro assays confirmed good cytocompatibility with osteoblast-like cells, along with promotion of cell adhesion and proliferation on the fibrous scaffold. Cell viability tests showed that, after 24 h, the cells maintain their integrity and spread throughout the membrane. Hence, the incorporation of BGNs in the polymeric blend has no negative effects on the cell attachment and proliferation, in comparison control group, as shown in Fig. 18.

**Fig. 19:**
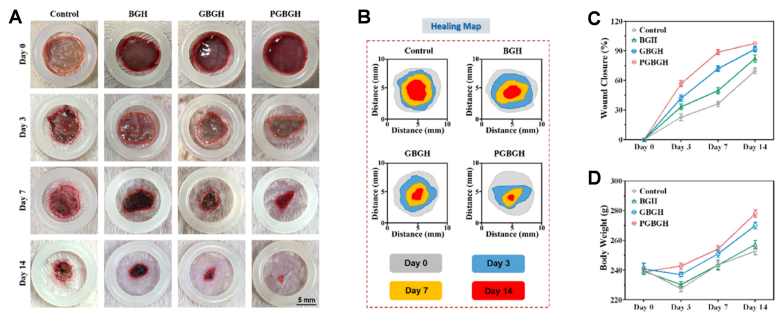
Wound healing assessment of the CS materials using MRSA-infected rats. (A) Representative photographs of MRSA-infected wounds at different times during the treatment, (B) schematic diagram of wound closure traces throughout the entire treatment process, (C) Quantitative analysis of wound closure area from day 0–14 in rats treated with different hydrogels, (D) the weight change of rats in each group during the whole treatment period. Reprinted and adapted with permission from Ref. [158]. Copyright 2026 Elsevier.

Jian et al. [158] developed an injectable nanocomposite hydrogel based on an allyl glycidyl ether grafted gelatin (Gel-AGE) and cysteamine grafted hyaluronic acid (HA-CSA) matrix, reinforced with CS nanosystems based on poly(procatechualdehyde) (PPA)-coated Ga-doped BGs (75 % SiO_2_ – 16 % CaO – 4 % P_2_O_5_ – 5 % Ga) (mol) for scarless healing of infected wounds. The core material was prepared via sol-gel in ethanol solution using CTAB, TEP, Ca(NO_3_)_2_•4H_2_O , TEOS, Ga(NO_3_)_3_•H_2_O, and NH_4_OH, under stirring for 3 h. The material was calcined at 650 °C for 3 h. The CS material was synthesized via in situ polymerization with the core materials being dispersed in water alongside procatechualdehyde and left under stirring for 12 h in the dark, followed by drying at 60 °C for 12 h. Release assays showed the PPA shell effectively inhibited the burst release of Ga^3+^ ions, which improved the hydrogel's biocompatibility. while maintaining excellent antibacterial activity against both MRSA and *E. coli*. The hydrogel also exhibited significant antioxidant properties, improved mechanical strength, and enhanced tissue adhesion due to the formation of Schiff base bonds between the PPA coating and skin tissue. Biological assays revealed that the hydrogel promoted the polarization of macrophages toward the anti-inflammatory M2 phenotype. In an in vivo MRSA-infected wound model in rats, the hydrogel significantly promoted angiogenesis, granulation tissue formation, and re-epithelialization, leading to faster wound closure, as shown in Fig. 19.

Organic and inorganic shells play distinct yet complementary roles in the design of CS nanomaterials. Inorganic shells, such as silica, zirconia, or copper, provide mechanical robustness, thermal stability, and tunable porosity, which support controlled drug loading and sustained release. They can offer antimicrobial activity depending on their composition. On the other hand, organic shells, including natural polymers like chitosan and alginate or synthetic ones like PLGA and PNIPAM, contribute biocompatibility, biodegradability, and stimuli-responsiveness, enabling targeted and responsive delivery. Furthermore, polymeric shells can improve dispersibility, prolong circulation time, and modulate immune interactions. Table 2 summarizes the main differences between these two types of materials.

**Table 2:** Comparison of Inorganic vs. Polymeric Shells.

Feature	Inorganic Shells	Organic/Polymeric Shells
Primary Advantages	**Robustness** (Mechanical, Thermal, Chemical) **Stability**: Low degradation, chemical inertness **Specific functions**: Magnetic (Fe_3_O_4_), antimicrobial (CuO, ZnO), osteoconductive (HA)	**Biocompatibility** and **Biodegradability** **Design Flexibility**: High tunability **"Smart" Functionality**: Stimuli-responsive (pH, Temp) **ECM Mimicry** (natural polymers)
Impacts on Mechanical Properties	Provides **hardness**, **robustness**, and **integrity** Used to improve **corrosion resistance** and **adhesion** of coatings Improves **mechanical resistance** Used as component in rigid composites	Improves properties of **composite systems** (scaffolds, hydrogels) Can provide **elasticity** and **stretchability** Improves **tensile strength** of fiber scaffolds
Release Modulation	Primarily a **physical diffusion barrier** Release is controlled by shell thickness and porosity Can be engineered to be porous to allow ion diffusion from the core	Release controlled by **diffusion** AND **shell degradation** Enables "on-demand" release via stimuli-response
Biological Interaction	Often biologically **inert** Bioactivity relies on specific shell material or ions from the core Antimicrobial properties	Often **bioactive**; can **mimic ECM**, promote cell adhesion Intrinsic properties Degrades into (ideally) biocompatible byproducts
Limitations & Scalability challenges	Lack of biodegradability or bioactivity is common A dense shell can overly inhibit ion release and bioactivity from the BGN core Synthesis can require harsh conditions	Lower mechanical/thermal robustness than inorganics Polymer degradation byproducts (e.g., acids from PLGA) can alter core stability Variability in natural polymers (batch-to-batch)
HA – hydroxyapatite, ECM – extracellular matrix, PLGA – polylactic-co-glycolic acid.		

Thus, these shells can be tailored to modulate release kinetics, enhance biological performance, and expand the versatility of BG-based CS systems for a wide range of therapeutic applications. Table 3 summarizes the examples presented and compares the core and shell compositions.

**Table 3:** Comparative summary of core–shell nanostructures with BGNs/MBGNs cores.

Core						Shell						
Material	Composition (mol)	Size (nm)	Pore Size (nm)	SSA (m^2^/g)	Pore Volume (cm^3^/g)	Composition	Thickness (nm)	Fabrication method	Drug,LE/EE	In vitro/In vivo models	Advantages	Ref
Binary BGNs	70 % SiO_2_ – 30 % CaO	118 ± 9	N.A.			ZrO_2_	16 ± 4	Sol-gel	N.S.	Bioactivity in SBF	Improved corrosion resistance and biocompatibility	[119]
	85 % SiO_2_ – 15 % CaO	50				Chitosan	5–19	Self-assembly	Phenamil/dexamethasone/FGF2, paclitaxel/doxorubicin	Macrophage cells (RAW264.7) – anti-inflammatory, Endothelial cells (HUVECs) – pro-angiogenic, Mesenchymal stem cells (MSCs) - osteogenic, Rat calvarium defect	Nanoroughness, Controlled sequential release of multiple therapeutic, Pro-angiogenic and osteopromoting	[44]
	70 % SiO_2_ – 30 % CaO	118 ± 9				ZrO_2_	16 ± 4	Sol-gel	N.S.	Bioactivity in SBF, Osteoblast-like (MG-63) - viability	Uniform coatings, Enhanced adhesion on metallic implants	[116]
Ternary BGNs	65 % SiO_2_ – 25 % CaO – 10 % MgO	300				CuO	2–10	Ion exchange	N.S.	Methicillin-resistant *S. aureus* (MRSA) – antibacterial, Macrophages (RAW 264.7) – immunomodulation, Fibroblasts (MC3T3-E1) – proliferation, Full-thickness wound model infected with MRSA in mice	Controlled sequential release of Cu^2+^ and Mg^2+^, Promotes specific immunomodulation, Accelerates wound closure in vivo.	[120]
	80 % SiO_2_ – 15 % CaO – 5 % P_2_O_5_	N.S.				PMMA	201	Polymerization	N.S.	Bioactivity in SBF, Osteoblast-like (MG-63) and fibroblast (L929) – viability	Mechanical reinforcements, biocompatibility	[153]
	55 % SiO_2_ – 15 % CaO – 30 % P_2_O_5_	70 ± 15				PCL/gelatin/PVDF/PVA	N.S	Electrospinning	Doxycycline	Bioactivity in SBF, Platelet adhesion, red blood cell (RBC) hemolysis and blood coagulation – hemocompatibility, *E. coli* and *S. aureus* – antibacterial, fibroblasts (L292) – viability	Controlled drug release, Antibacterial Activity, Hemocompatibility	[157]
	70 % SiO_2_ –20 % CaO −10 % SrO	N.S.				CuO	12	Sol-gel	N.S.	Macrophages (RAW 264.7) – immunomodulation, Mesenchymal stem cells (rBMSCs) – osteogenesis, Methicillin-resistant *S. aureus* (MRSA) – antibacterial, Subcutaneous implant in mice (C57BL/6) – immunomodulation, MRSA-infected rat femur model	Controlled sequential release of Cu^2+^ and Sr^2+^, Osteogenesis, Antibacterial activity, Immunomodulatory effect	[121]
Quaternary BGNs	40 % SiO_2_ – 24.5 % Na_2_O – 30 % CaO – 5.5 % P_2_O_5_	20				Chitosan	370–660	Layer-by-layer coating	N.S.	Ca^2+^ release in water	Tunable calcium release, Prolonged bioactivity	[138]
Quinary BGNs	40 % SiO_2_ – 10 % Fe_2_O_3_ – 10 % Li_2_O – 36 % CaO – 4 % P_2_O_5_	55–80				Hydroxypropyl cellulose	50–60	Coating	Doxorubicin	Bioactivity in SBF, Hyperthermia, Human osteosarcoma (G292) – cytotoxicity	Chemo-hyperthermia properties, biocompatibility	[141]
Binary MBGNs	85 % SiO_2_ – 15 % CaO	116	N.S			PNIPAM	15	Polymerization	N.S.	Ca^2+^ release in water	Temperature-responsiveness	[154]
Ternary MBGNs	80 % B_2_O_3_ –10 % CaO – 10 % Li_2_O	40–50	N.S			HA	N.S	Deposition	N.S.	Bioactivity in SBF, Human Dental Pulp Cells (hDPCs) – cytotoxicity and osteogenic differentiation, *E. coli*, *S. aureus* and *S. mutans* – antibacterial	Direct pulp capping applications, Antibacterial properties	[122]
	60 % SiO_2_ – 36 % CaO – 4 % P_2_O_5_	40 ± 12	N.S.	183.95	0.12	poly (lactic-co-glycolic acid)	N.S.	electrospinning	5-Fluorouracil	Fibroblasts (L929) – biocompatibility, cell migration, Melanoma cells (B16) – cytotoxicity, Full-thickness skin wound model in mice (C57BL/6), Partial (90 %) and complete resection models of melanoma in C57BL/6 mice	Wound healing, Controlled release	[155]
	80 % SiO_2_–15 % CaO–5 % P_2_O_5_	170.87	10.46	356	0.47	poly-L-glutamic acid	N.S	polymerization	LE 26.82 % EE 93.85	*P. aeruginosa* and *B. subtilis* – antibacterial	pH-responsiveness	[156]
Quaternary MBGNs	55 % SiO_2_ – 30 % CaO – 10 % CoO – 5 % B_2_O_3_	180–250	N.S.	193	0.6	SiO_2_ + SPIONs	25	Sol-gel	N.S.	Mouse pre-osteoblasts (MC3T3-E1) and Osteoblast-like (MG-63) – biocompatibility, cellular mineralization, and angiogenesis/osteogenesis, DNA fragmentation assay	Superparamagnetism, No genotoxicity, Angiogenic effect	[123]
	75 % SiO_2_ – 16 % CaO – 4 % P_2_O_5_ – 5 % Ga	133 ± 4	N.S.			poly(protocatechualdehyde)	8	Polymerization	N.S.	Cells (RAW 264.7) and (NIH-3T3) – biocompatibility, cell migration, macrophage polarization and oxidative stress, Methicillin-resistant *S. aureus* (MRSA) and *E. coli* – antibacterial, Hemolysis in rat erythrocytes – Hemocompatibility, Full-thickness skin wound model in Sprague-Dawley rats infected with MRSA – healing, Rat hepatic hemorrhage model – hemostasis	Biocompatibility, Angiogenesis	[158]

SBF – Simulated Body Fluid; MRSA – Methicillin-resistant S. aureus (MRSA); RBC – red blood cell; ZrO2 – Zirconium oxide; CuO – Copper oxide; PMMA – Poly(methyl methacrylate); PCL – Polycaprolactone; PVDF – polyvinylidene fluoride; PVA – polyvinyl alcohol; PNIPAM – Poly(N-isopropylacrylamide); HA – Hydroxyapatite; SPIONs – Superparamagnetic iron oxide nanoparticles; N.S. – not specified; N.A. – not applicable; EE – encapsulation efficiency; LC – loading capacity.

## 5 Challenges and future perspectives

Despite their significant potential in biomedical applications such as tissue regeneration and engineering, CS nanostructures that use BGNs as the core still present several challenges in their design, fabrication, and translation to clinical use. One of the significant limitations is the complexity of synthesis, particularly in achieving uniformity, reproducibility, and scalability. The methods commonly reported in the literature (sol–gel coating, layer-by-layer assembly, or in situ polymerization) generally require precise control over parameters such as shell thickness, porosity, and homogeneity to ensure consistent performance [159,160]. In addition, designing these systems in a way that maintains bioactivity and enables controlled release of ions and therapeutic agents requires a delicate balance. The interactions between the core and shell can affect both the release profile and the structural integrity of the nanosystems. For example, acidic degradation products from polymeric shells may alter the reactivity or stability of the core [44,157]. Another related challenge is achieving spatiotemporal control over the release of drugs and ions, especially in complex physiological environments, which requires advanced functionalization strategies and targeted or stimulus-responsive systems. These requirements are strictly linked to the need for homogeneity and precise control over the materials’ structure [44,161,162]. In addition to these difficulties, it is worth mentioning that the formation of composite shells is a major challenge. Even though they can offer synergistic properties, obtaining homogeneous structures can present difficulties.

Other challenges are also introduced regarding the biological performance of CS nanosystems. Although BGNs have consistently demonstrated their biocompatibility and regenerative properties, the long-term in vivo behavior of hybrid systems using these BGNs remains still underexplored. The availability of long-term biological compatibility assays is limited, since the vast majority of studies focus on short-term cytocompatibility or in vitro drug release, which are unable to accurately predict the performance of the material in complex physiological environments [44,163]. Thus, it is still necessary to obtain a deeper understanding and comprehensive evaluation of the degradation kinetics, immune response, and bioabsorption of the shells, especially for synthetic and inorganic shells, not only of the intact nanosystem but also of its degradation products. For inorganic shells, the concern lies in their stability and fate. Highly stable shells may resist degradation, leading to potential bioaccumulation in reticuloendothelial system organs, such as liver and spleen, for which the chronic toxicity threshold is unknown. Conversely, shells that do degrade release ionic species beyond those from BGN core [164,165]. Despite presenting beneficial properties, the effect of long-term bioaccumulation of some ionic species should be better explored to avoid potential inflammatory responses or undesired tissue responses. Regarding BGNs themselves, especially those obtained via sol-gel mthods, the risk of a burst release of ions in the initial phases can cause localized pH spikes or ionic overload, an effect that is often buffered in vivo but poorly understood in the long term. In polymeric shells, the same can be said for oligomers that can be released during hydrolysis. These byproducts can act as haptens or directly modulate the innate immune response, interacting with immune cells, influencing macrophage polarization, cytokine expression, or fibrotic encapsulation [166]. Therefore, pharmacokinetic and biodistribution studies, using appropriate animal models and with extended follow-up times (months), are essential not only to optimize nanoparticle dose, administration route, and therapeutic window but also to quantify the clearance of accumulation of nano-hybrid components in key organs and evaluate the local and systemic tissue response over time [164,167]. From a translational point of view, approval by regulatory agencies for large-scale production of these multifunctional nanomaterials faces significant obstacles generated by reproducibility, cost, and compliance with safety standards [19,104].

Still regarding the biological performance of CS nanosystems, the establishment of a clear quantitative correlation between ion release and biological response remains as one of the major challenges in the study of BGN-based systems. Although several studies have demonstrated the osteogenic, angiogenic, and antibacterial effects of ions such as Ca^2+^, Cu^2+^, and Zn^2+^, the specific concentration ranges required to trigger these effects remain undefined [[168], [169], [170]]. This limitation arises primarily from the intrinsic complexity of BG matrices, which simultaneously release multiple ionic and molecular species acting in a synergistic and interdependent manner. Such interactions make it extremely difficult to isolate the contribution of individual ions and to define precise therapeutic thresholds. Moreover, each CS configuration presents distinct dissolution behaviors depending on its composition, porosity, and shell permeability, which leads to variations in the ion release kinetics and consequently in the biological outcomes. Some approaches show that mathematical and computational models are being developed to predict multi-ionic release and biological outcomes, but experimental validation remains limited [171,172]. Therefore, future investigations should aim to integrate systematic dose–response studies with quantitative modeling of multi-ionic release profiles, providing a deeper understanding of how these dynamic processes govern the biological performance of BGN-based CS nanostructures [38].

Looking ahead, these next-generation CS systems have great potential to integrate multifunctionality, real-time imaging capabilities, responsive behavior to biological, and external stimuli, and even to combine therapeutic responses (such as simultaneous osteogenic, anti-microbial, anti-inflammatory and angiogenic properties) [45,173]. Future advances in underexplored technologies for these materials, such as microfluidic synthesis, machine learning- and artificial intelligence-based design optimization, and even bioprinting, may provide the necessary tools for the precise and scalable fabrication of these complex nanostructures. Furthermore, the development of standardized biological models and *in vivo* assay protocols is essential to bridge the current gap between proof-of-concept studies and future clinical translation. As the understanding of the structure-property-functionality relationships of these systems deepens, BGN-based CS nanomaterials are moving closer to becoming highly tunable platforms for personalized and regenerative nanomedicine as revealed by the outcomes of the studies discussed in this review [19,45,104,173].

## 6 Conclusion

CS nanoparticles incorporating BGN cores represent a very recent and significant advance in the development of nanocarriers for biomedical applications. BGNs present advantages in comparison to conventional silica due to their intrinsic bioactivity and ability to release therapeutic ions such as Ca^2+^, Sr^2+^, Cu^2+^, and Zn^2+^, which are known to stimulate osteogenesis, angiogenesis, immunomodulation, biomineralization, and antibacterial responses. This review has highlighted how the rational design of CS systems combining BGN cores with meticulously engineered inorganic shells, like zirconia or HA, can enhance mechanical strength, mineralization and photothermal activity. On the other hand, polymeric shells (e.g., chitosan, alginate, PEG, PLGA) provide improved biocompatibility and responsiveness to stimuli (pH, temperature), enabling controlled and targeted drug delivery. These hybrid systems offer a versatile platform for merging regenerative and therapeutic functionalities.

Future developments should further expand these capabilities through multi-functional coating, co-loading of bioactive molecules or integration with smart, stimuli-responsive systems for precision therapies. However, several challenges remain: limited *in vivo* and clinical data, the complexity of large-scale reproducible fabrication, and the need for deeper understanding of degradation kinetics, immune response, and long-term safety.

In summary, BGN-based CS nanoparticles have proven to be highly adaptable platforms, with high synergy and the possibility of merging regenerative and therapeutic functions. Continued advances in biomaterial nanofabrication and biological evaluation are contributing to the expansion of applications of these nanosystems, which can become important players in the future of personalized and regenerative nanomedicine.

## CRediT authorship contribution statement

**Arthur M. Gabriel:** Writing – review & editing, Writing – original draft, Visualization, Validation, Project administration, Methodology, Investigation, Formal analysis, Data curation, Conceptualization. **Andrada-Ioana Damian-Buda:** Writing – review & editing, Visualization, Validation, Data curation. **Fernanda M. Brugnari:** Writing – original draft, Validation, Data curation. **Emerson R. Camargo:** Writing – review & editing, Supervision, Resources, Project administration, Investigation, Funding acquisition, Conceptualization. **Aldo R. Boccaccini:** Writing – review & editing, Visualization, Validation, Supervision, Resources, Project administration, Investigation, Funding acquisition, Conceptualization.

## Declaration of generative AI and AI-assisted technologies in the writing process

During the preparation of this work, the authors used ChatGPT in order to improve the readability and language. After using this tool/service, the authors reviewed and edited the content as needed and take full responsibility for the content of the published article.

## Declaration of competing interest

The authors declare the following financial interests/personal relationships which may be considered as potential competing interests: Arthur Martins Gabriel reports financial support was provided by State of Sao Paulo Research Foundation. Emerson Rodrigues de Camargo reports financial support was provided by State of Sao Paulo Research Foundation. Emerson Rodrigues de Camargo reports financial support was provided by National Council for Scientific and Technological Development. Emerson Rodrigues de Camargo reports financial support and article publishing charges were provided by Coordination for the improvement of Higher Education Personnel. If there are other authors, they declare that they have no known competing financial interests or personal relationships that could have appeared to influence the work reported in this paper.

## Data Availability

No data was used for the research described in the article.
